# Exercise for prevention of falls and fall-related injuries in neurodegenerative diseases and aging-related risk conditions: a meta-analysis

**DOI:** 10.3389/fendo.2023.1187325

**Published:** 2023-07-14

**Authors:** Feifei Feng, Haocheng Xu, Yu Sun, Xin Zhang, Nan Li, Xun Sun, Xin Tian, Renqing Zhao

**Affiliations:** ^1^ College of Physical Education, Yangzhou University, Yangzhou, China; ^2^ School of Humanities and Education, Guangzhou Nanyang Polytechnic College, Guangzhou, China

**Keywords:** falls, fall-related injuries, neurodegenerative diseases, aging, exercise

## Abstract

**Introduction:**

Neurodegenerative diseases often cause motor and cognitive deterioration that leads to postural instability and motor impairment, while aging-associated frailty frequently results in reduced muscle mass, balance, and mobility. These conditions increase the risk of falls and injuries in these populations. This study aimed to determine the effects of exercise on falls and consequent injuries among individuals with neurodegenerative diseases and frail aging people.

**Methods:**

Electronic database searches were conducted in PubMed, Cochrane Library, SportDiscus, and Web of Science up to 1 January 2023. Randomized controlled trials that reported the effects of exercise on falls and fall-related injuries in neurodegenerative disease and frail aging people were eligible for inclusion. The intervention effects for falls, fractures, and injuries were evaluated by calculating the rate ratio (RaR) or risk ratio (RR) with 95% confidence interval (CI).

**Results:**

Sixty-four studies with 13,241 participants met the inclusion criteria. Exercise is effective in reducing falls for frail aging people (RaR, 0.75; 95% CI, 0.68–0.82) and participants with ND (0.53, 0.43–0.65) [dementia (0.64, 0.51–0.82), Parkinson’s disease (0.49, 0.39–0.69), and stroke survivors (0.40, 0.27–0.57)]. Exercise also reduced fall-related injuries in ND patients (RR, 0.66; 95% CI, 0.48–0.90) and decreased fractures (0.63, 0.41–0.95) and fall-related injuries (0.89, 0.84–0.95) among frail aging people. For fall prevention, balance and combined exercise protocols are both effective, and either short-, moderate-, or long-term intervention duration is beneficial. More importantly, exercise only induced a very low injury rate per participant year (0.007%; 95% CI, 0–0.016) and show relatively good compliance with exercise (74.8; 95% CI, 69.7%–79.9%).

**Discussion:**

Exercise is effective in reducing neurodegenerative disease- and aging-associated falls and consequent injuries, suggesting that exercise is an effective and feasible strategy for the prevention of falls.

## Introduction

Falls and subsequent injuries contribute to a major health problem for older people worldwide (1–4). It has been estimated that one-third of community-dwelling people aged 65 years and over have at least one fall each year (5, 6). Falls can have serious consequences, such as fractures and head injuries (7). Approximately 10% of falls result in serious injuries contributing to a major source of morbidity and mortality (5, 6, 8).

Aging and neurodegenerative diseases are two deleterious conditions that increase the risk of falls and subsequent injuries (9–13). Frailty represents a decline in multiple systems due to a mixture of physiological and psychological factors, such as low muscle mass and strength, slow walking speed, functional impairment, and weakness (14, 15). Frailty caused by aging often results in lower leg muscle strength (16, 17), poor balance and posture control (18, 19), and lack of flexibility (20), which raises the risk of falling for older people. The risk factors for falls are worsened in neurodegenerative diseases. Impaired gait, balance, and posture are common symptoms of dementia, Parkinson’s disease, and stroke. These symptoms impair posture control and motor capacity, which greatly elevate the fall risk (21, 22). For example, stroke survivors with serious impairment in gait and balance have a much higher risk of falling than healthy adults of a similar age, showing nearly seven times more fall rates (23). Since those people have to confront increased falling and fall-related injuries in their daily lives, developing effective strategies to prevent falls and subsequent injuries is an emergent task.

Exercise has been recognized as an effective approach to fall prevention; it targets improving or maintaining muscle mass and strength, bone mass, balance and posture control, gait, and other aspects of physical functioning (18, 24–27). Therefore, strategies that aim to modify those risk factors are recommended for preventing falls and subsequent injuries (28). To date, several meta-analyses and Cochrane Reviews (29–31) have evaluated the beneficial effects of exercise in the prevention of falls in healthy community-dwelling people. However, it is still unknown whether exercise could modify the risk factors and reduce falls and injuries in frail aging people and individuals with neurodegenerative diseases. Addressing the question is important because falls and serious injuries are more likely to occur in those high-risk people. Given the fact that the exact aspects of the exercise regimens such as intervention effects, safety, and complication of exercise have not been evaluated, it turns out to be a challenge both for those individuals and their physicians in determining whether to choose exercise as a strategy for fall prevention. To address those concerns, we conducted a systematic review and meta-analysis to determine the effect of exercise on falls and subsequent injuries in frail aging people and individuals with neurodegenerative diseases (stroke, dementia, and Parkinson’s disease). The efficacy, safety, and compliance of exercise protocols were also evaluated.

## Methods

### Search strategy and inclusion criteria

This systematic review and meta-analysis adhered to the Preferred Reporting Items for Systematic Reviews and Meta-Analyses (PRISMA) guidelines (32). This meta-analysis was not registered. The search strategies for electronic databases are listed in **Supplementary Texts S1–S4**. Two authors (CX and YS) conducted the electronic database searches in PubMed, Cochrane Library, SportDiscus, and Web of Science using a predetermined protocol up to 1 January 2023. After the removal of duplicates, the articles were screened on the basis of titles and abstracts, and another author (XZ) reviewed them independently. The full text of the articles then was drawn and reviewed for eligibility independently by BZ and CZ. To identify unpublished studies, the WHO International Clinical Trials Registry Platform (ICTRP) search portal and ClinicalTrials.gov were searched up to 1 January 2023. The reference lists of the relevant systematic reviews were screened manually to identify further potentially relevant citations. No language restriction was applied.

Eligible studies should meet the following criteria: (1) randomized controlled trials (RCTs) that investigated the effects of exercise on the rate of falls; (2) studies compared exercise intervention group with a comparator group, such as no intervention, attention, or sham exercise (e.g., light physical activities), which was expected to have no effects on muscle strength, balance, or mobility, etc.; (3) if studies compared several groups (e.g., nutrition supplementations, placebo, and exercise groups), the groups with placebo and exercise interventions were considered for inclusion; and (4) participants aged ≥ 60 years (mean or median age) and either had neurodegenerative diseases or aging-associated frailty. The included participants who had neurodegenerative diseases such as dementia, Parkinson’s disease, and stroke were not particularly restricted by the subtypes and stages of the diseases, but they needed to have the ability to perform the exercise protocol. Frail aging people included in the original studies had some of the conditions at enrollment that predisposed participants to be at high risk of falling, including lower limb weakness, impaired balance, slow reaction time, a history of falling, and other functional deficits. The common community-dwelling older people without those obvious health conditions mentioned above were not included in this review and have been addressed elsewhere (29–31, 33).

### Outcome measures

Outcomes were as follows:

- Primary outcomes: the rate of falls (falls per person-year).
- Secondary outcomes: (1) rate of fall-related injuries (injuries caused by a fall, e.g., fall-induced wound, head trauma, medical care, or hospitalization, according to original investigators); (2) number of people who sustained one or more fractures.

### Study selection and data collection

Two authors (CX and YS) performed data abstraction independently using a standard collection data form designed for this meta-analysis. In cases of disagreement, an agreement was achieved through discussion with another author (XZ). If insufficient data were presented in the original studies, we contacted investigators for detailed information. The following data were extracted: sample size, participant age, countries, study design, exercise interventions (category, intensity, frequency, and duration), participant health conditions, attrition, exercise compliance, exercise-related injuries, length of follow-up, number or rate of falls, fall-related injuries, and fractures.

### Risk of bias assessment

The risk of bias assessment for each included study followed the recommendations of the Cochrane Collaboration (34). For each trial, pairs of members of the review team (CX and YS) reported the following key domains: sequence generation, allocation concealment, blinding, incomplete outcome data, and selective reporting. Each domain was judged to be low, unclear, or high risk of bias. The final assessment for all studies was presented in a “risk of bias” table.

### Statistical analysis

We reported the treatment effects for rate of falls as rate ratio (RaR) with 95% confidence interval (CI). For fall-induced injuries and the number of participants experiencing fractures, we reported risk ratio (RR) and 95% CI. We used results reported at the last measurement for trials that monitored falls, fall-related injuries, and fractures during follow-up. We used RaR or hazard ratio (HR) with 95% CI if these were reported in the trials. If RaR was not reported, but appropriate raw data were available, we calculated it using the reported rate of falls in each group, or we calculated the rate of falls in each group from the total number of falls and the actual total length of time were monitored (person-years) for participants contributing data. We used reported estimates of RR or HR with 95% CI for fractures if available. If RR was not reported and appropriate data were available, we calculated RR and 95% CI using STATA software. Random-effects models were used for calculating RaR for falls and evaluating RR for fall-induced injuries and fractures.

We first calculated the summary effects of exercise on falls, fall-related injuries, and fractures in frail aging people and participants with neurodegenerative diseases (dementia, Parkinson’s disease, and stroke), respectively. Subgroup analysis was conducted to estimate the intervention effects on falls by grouping exercise categories (combined exercise, strength training, balance training, and aerobic exercise) and intervention durations (<6 months, 6–12 months, and ≥12 months), respectively.

We used Cochran’s Q test (p<0.1 considered significance) and I^2^ (<40%, 40%–<75%, and ≥75% for low, moderate, and high heterogeneity) to assess between-study heterogeneity. The test for the overall effects (z score) was regarded as significant at p<0.05. STATA version 15 software (StataCorp LP, College Station, TX, USA) was used for performing a meta-analysis.

### Sensitivity and publication bias analyses

We carried out a sensitivity analysis to explore the possible impact of risk of bias on pooled estimates of treatment effects. Trials with high risk or unclear bias in domains were removed from the analysis, and sensitivity analysis was performed for falls, fall-related injuries, and fractures among frail aging people and participants with neurodegenerative diseases. To determine publication bias, we constructed funnel plots for falls, fall-related injuries, and fractures with treatment effects (RaR/RR) estimated from individual studies against a measure of study size (standard error of log RaR/log RR). We used Egger’s test to examine the likelihood of the presence of small-study effects.

## Results

### Study selection

The electronic database searches identified and screened 4,173 abstracts, of which 4,075 were excluded because of either unrelated to the topic (n=2,878) or duplicate studies (n=1,197). A total of 98 full-text articles were reviewed for eligibility, and 43 studies were excluded for the reasons: (1) no reporting data of interest (n=16), (2) no RCTs (n=15), (3) no exercise intervention group (n=7), (4) wrong age (n=2), and (5) no sedentary controls (n=3). A total of 55 studies were eligible for meta-analysis. We also identified nine new trials from previous meta-analyses. Therefore, 64 RCTs in total met the inclusion criteria (**Supplementary Figure S1**, **Supplementary eText S5**).

### Characteristics of included

Details are provided in the characteristics of included studies (**Supplementary eTable S1**). Due to the size of the meta-analysis, not all links to references have been inserted in the following text, which can be viewed in **Supplementary eText S5**. Briefly, 64 RCTs with 13,241 participants (neurodegenerative diseases, 2,539; aging, 10,702) were included, of which 7,040 received exercise intervention and 6,201 complied with the requirements for controls. The characteristics of participants included neurogenerative diseases (stroke, dementia, and Parkinson’s disease) and aging-related frailty (**Supplementary eTable S1**).

The included studies were conducted in 19 countries, the most common countries being Australia (13 trials), the USA (eight trials), Finland (six trials), the UK (eight trials), China (four trials), Japan (four trials), Germany (three trials), France (three trials), and Canada (three trials). The remaining studies were carried out in the Netherlands (two trials), Sweden (two trials), New Zealand (one trial), Brazil (one trial), Hungary (one trial), Denmark (one trial), Iran (one trial), Portland (one trial), Spain (one trial), and Thailand (one trial) (**Supplementary eTable S1**).

Four types of exercise programs were detected in the included studies, with 42 studies involving combined exercise, 19 performing balance training, 3 conducting strength training, and 1 carrying out aerobic activities. Combined exercise protocols frequently include several distinct types of exercise (such as strength training, balance exercise, aerobic activities, and impact exercises), which are incorporated into one training class to augment the intervention effects (35, 36). Exercise interventions were delivered in centers (28 studies), homes (16 studies), or centers and homes (20 studies).

### Study quality assessment

We applied a widely used methodological quality assessment tool (34) to evaluate the quality of individual studies. Of the 64 included trials, 27 studies scored low risk for all domains, 21 studies marked at least one high-risk domain, and 16 studies marked at least one unclear risk in domains (**Supplementary eTable S2**). The most common domain that scored a high risk of bias was selective reporting. Blinding in many trials was marked unclear risk, and it was used for outcome assessment but not for participants.

### Meta-analysis of neurodegenerative diseases

**Figure 1:**
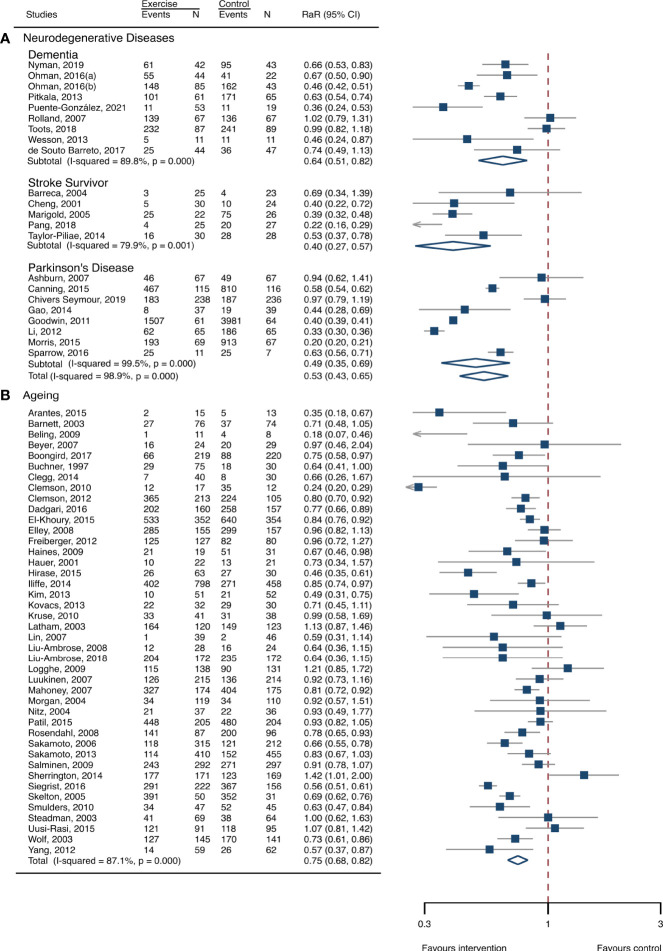
Summary estimates of exercise for prevention of falls in participants with neurodegenerative diseases and frail aging people. **(A)** Participants with neurodegenerative diseases; **(B)** frail aging people. RaR, rate ratio; CI, confidence interval; N, number.

**Figure 2:**
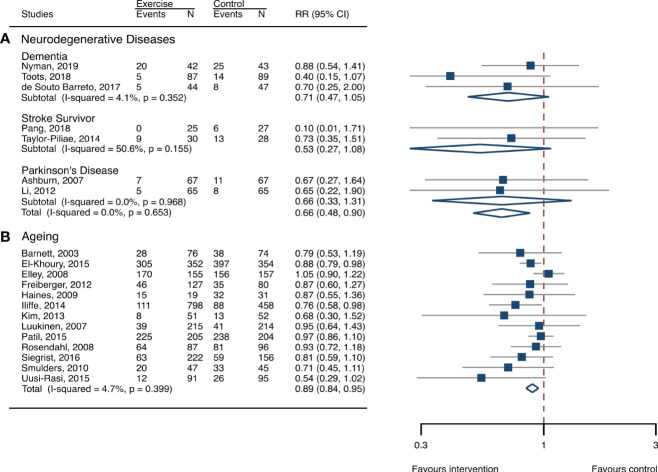
Effects of exercise on the risk of fall-related injuries. **(A)** Participants with neurodegenerative diseases; **(B)** frail aging people. RR, risk ratio; CI, confidence interval; N, number.

**Figure 3:**
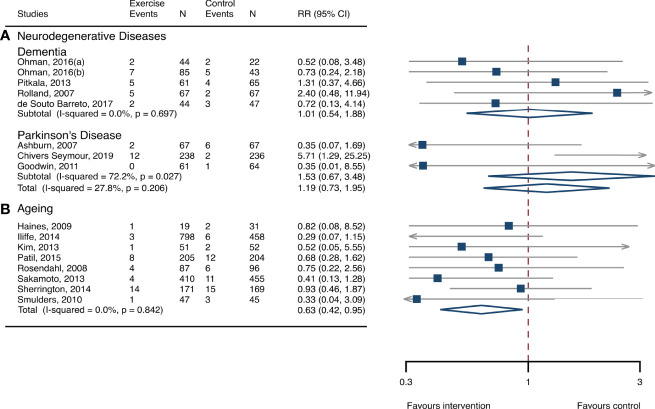
Effects of exercise on the risk of fractures. **(A)** Participants with neurodegenerative diseases; **(B)** frail aging people. RR, risk ratio; CI, confidence interval; N, number.

**Figure 4:**
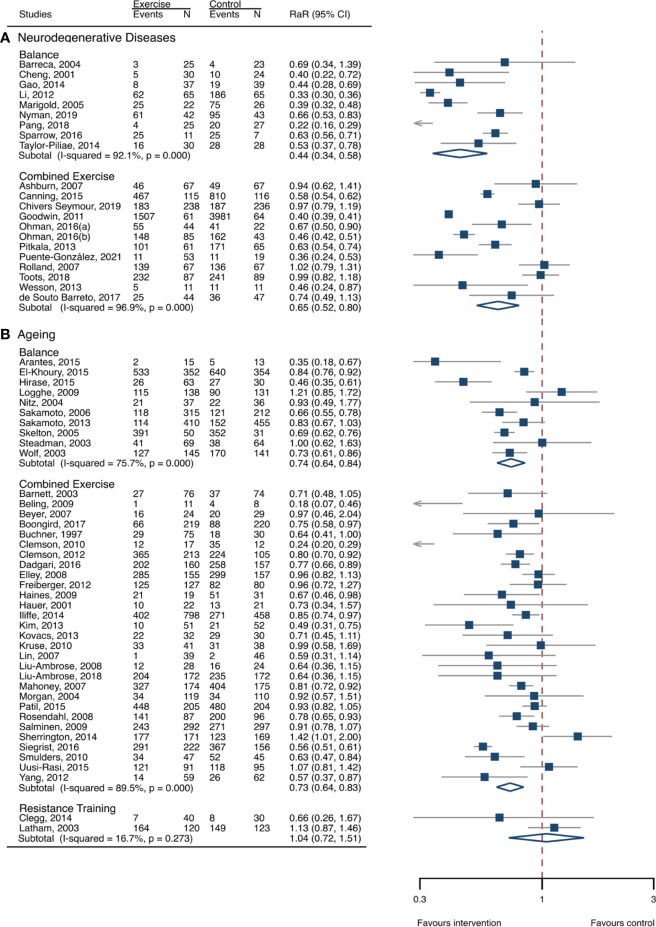
Effects of exercise on fall rates categorized by exercise types. **(A)** Participants with neurodegenerative diseases; **(B)** frail aging people. RaR, rate ratio; CI, confidence interval; N, number.

**Figure 5:**
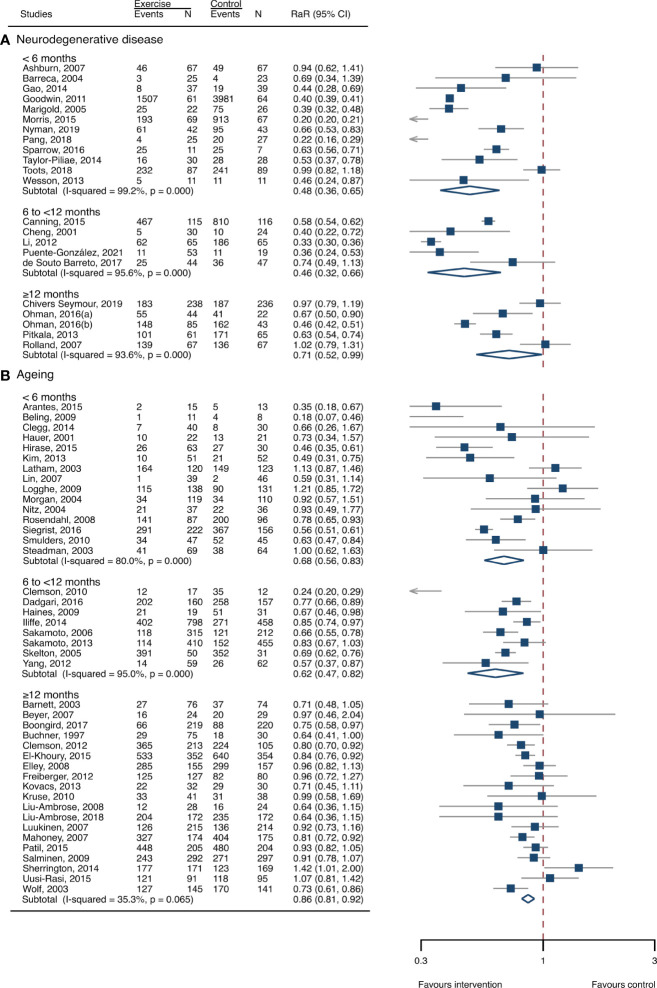
Effects of exercise on fall rates categorized by exercise durations. **(A)** Participants with neurodegenerative diseases; **(B)** frail aging people. RaR, rate ratio; CI, confidence interval; N, number.

The between-study heterogeneity was high (I^2^ > 75%) for fall rates and was low (I^2^ < 30%) for fractures and fall-related injuries (**Figure 1**). Exercise generated an overall reduction in fall rates for participants with neurodegenerative diseases (RaR, 0.53; 95% CI, 0.43–0.65). Among the participants, exercise reduced rates of falls for dementia by 36% (0.64, 0.51–0.82), stroke by 60% (0.40, 0.27–0.57), and Parkinson’s disease by 51% (0.49, 0.39–0.69) (**Figure 1**). Exercise also reduced fall-related injuries in participants with neurodegenerative diseases (RR, 0.66; 95% CI, 0.48–0.90) (**Figure 2**) but did not generate beneficial effects on fracture prevention (1.19, 0.73–1.96) (**Figure 3**). Subgroup analysis suggested that both combined and balance training protocols were effective in reducing fall rates, with RaR of 0.65 (0.52–0.80) and 0.44 (0.34–0.56), respectively (**Figure 4**). Additionally, for fall prevention, any training durations (<6, 6–<12, and ≥ 12 months) are beneficial, with intervention effects 0.48 (0.36–0.45), 0.46 (0.32–0.66), and 0.71 (0.52–0.99), respectively (**Figure 5**). More importantly, the exercise regimens only generated a low exercise-induced injury rate per participant year (0.005, 95% CI 0–0.02) and showed good compliance with exercise (78.4%; 95% CI, 66.9%–89.8%).

Although the funnel plots for falls showed that there existed somewhat asymmetry (**Supplementary Figure S2**), Egger’s test suggested that there existed weak evidence for the presence of small-study effects (p=0.068) (**Supplementary eTable S3**). The funnel plots for fall-related injuries and fractures seemed to exist asymmetry (**Supplementary Figures S3, S4**), but the likelihood of the appearance of small-study effects was weak for fractures and fall-related injuries (Egger’s test: p=0.151, p=0.018). Sensitivity assessment indicated that the intervention effects were still significant for falls (0.67, 0.48–0.93) and fall-related injuries (0.71, 0.51–0.98), and not changed significantly for fractures (1.33, 0.70–2.52) after removing the studies with either high or unclear risk of bias (**Supplementary eTable S4**).

### Meta-analysis of frail aging people

The heterogeneity across studies was relatively high (I^2^ > 75%) for falls and low (I^2^ < 5%) for fractures and fall-related injuries (**Figure 1**). The exercise was effective in preventing the rate of falls for frail aging people, resulting in a 25% fall reduction (RaR, 0.75; 95% CI, 0.68–0.82) (**Figure 1**). Exercise intervention also benefited the reduction in fractures and fall-related injuries, lowing the risk by 37% (RR, 0.63; 95% CI, 0.41–0.95) and 11% (0.89, 0.84–0.95), respectively (**Figures 2**, **3**). Subgroup analysis suggested that both combined and balance exercise regimens were effective in decreasing fall rates, with RaR of 0.73 (0.64–0.83) and 0.74 (0.64–0.84), respectively (**Figure 4**). Additionally, for fall prevention either short- (<6 months), moderate- (6–<12 months), or long- (≥12 months) term intervention durations are beneficial, with intervention effects of 0.68 (0.56–0.83), 0.62 (0.47–0.82), and 0.86 (0.81–0.92), respectively (**Figure 5**). Moreover, exercise intervention only induced a very low exercise-induced injury rate per participant year (0.008; 95% CI, 0–0.028) and show good compliance with exercise (73.0%; 95% CI, 66.1%–80.0%).

While the funnel plots for fall rates among frail aging people were relatively symmetry (**Supplementary Figure S5**), there existed a small asymmetry for fractures and fall-related injuries (**Supplementary Figures S6, S7**). However, Egger’s tests showed that there was weak evidence of the presence of small-study effects (p = 0.068 and p = 0.059) (**Supplementary eTable S3**). Sensitivity analysis suggested that the results remained significant for falls (0.85, 0.78–0.93) and fall-related injuries (0.91, 0.84–0.98), but not significant for fractures (0.71, 0.27–1.91) (**Supplementary eTable S4**).

## Discussion

Frequently, selecting exercise as a preventive strategy for reducing falls and injuries is a difficult choice for people with neurodegenerative diseases or frail aging adults who often confront balance instability and motor impairment. This meta-analysis has provided pooled evidence to confirm the beneficial effects of exercise on reducing falls and fall-related injuries in people with neurodegenerative diseases and frail older people. Fall-prevention exercise successfully decreased fractures in people with aging-related frailty. Combined and balance exercises were the common protocols adopted by older people, and any length of intervention duration generated beneficial outcomes for fall prevention.

Falls are a prevalent and serious problem for people with neurodegenerative disease or aging-associated frailty, leading to disability, hospitalization, and mortality (21–23, 28). Doctors frequently encounter challenges in prescribing exercise protocols for those populations. They might wonder, for example, whether exercise is effective in the prevention of falls and subsequent injuries and whether it is safe for practice. Our findings indicated that exercise was effective in preventing falls and consequent injuries in those people. It has been recognized that exercise is an effective strategy for correcting the low muscle strength (16, 17), poor balance and posture control (18, 19), immobility (37), and impaired gait (38, 39), etc., all of which are common in patients with dementia, stroke, and Parkinson’s disease, and some in frail older people. The findings suggest that adverse components causing falls are correctable in those people. It is important because current reviews mainly evaluate overall effects among common “community living people” (26, 29, 33, 40), but the fact is that more falls and serious consequent injuries frequently occur among older people with risk conditions, such as neurodegenerative diseases and aging-associated frailty. Given the fact that the risk factors of falls are hard to be corrected by pharmacological approach among those high-risk people, exercise provides a promising preventive strategy. This review bettered our understanding of the clinical implications of applying exercise to prevent falls and injuries among older populations in high-risk conditions.

Our findings that combined exercise was one of the optimal exercise protocols for preventing falls agreed with previous reviews (26, 29, 31). The combined exercise incorporates a range of distinct types of exercise and frequently generates a combined improvement in muscle strength, balance function, mechanical loading, and other physical functions (27, 35, 36), all of which are determinants of falls. Therefore, combined exercise could serve as a feasible approach for reducing fall risk in frail aging people and patients with neurodegenerative disorders who can adhere to such exercise regimes. This is consistent with previous findings that combined exercise is a common and effective protocol for preventing falls among aging people (31, 41), and it is also used as a fall-prevention strategy for neurodegenerative disorders (42–45).

Impairment of balance is recognized as the most frequent and sensitive risk factor for predisposing falls and subsequent injuries. Multiple risk factors, such as aging, neurodegenerative diseases, immobilization, and decreased physical activities, potentially decrease the individual’s ability to balance and posture control. Balance exercise is effective in correcting those risk factors and then shows favorable changes in reducing falls in aging people (33, 40) and populations with neurodegenerative disorders (43, 46–48). The benefits of balance training in preventing fall risk were also corroborated by a Cochrane review in older people (31). Strength exercise, however, did not generate significant changes in fall prevention in frail aging people. This is in accordance with a previous study that revealed that the effects of strength exercise on fall risk were uncertain (31). It is indicated that strength training is less effective for the management of falls in frail aging people and participants with neurodegenerative disorders. Our findings further indicated that, for improving the poor status of aging-associated frailty and neurogenerative diseases, even a relatively shorter exercise program (<6 months) could generate favorable results. Therefore, those results suggested that exercise was a feasible strategy for managing adverse outcomes of the poor status of older people.

Evidence based on the included RCTs showed that fall-prevention exercise only generated a relatively low injury rate among frail aging people, but it is unclear in patients with neurodegenerative diseases due to the lack of injury data. One population-based study (49) conducted a 24-month exercise program with 352 exercising older people, and only reported 4 injuries during the intervention and another study (50) on aging populations reported no injury during training. However, only one study (51) reported exercise-related injuries among a population of 231 Parkinson’s disease participants and revealed two injuries during exercising time. The compliance of exercise was relatively good for both aging people and participants with neurodegenerative disorders. Two included studies (52, 53) reported the percentage of exercise compliance of approximately 84% and 79% for older people and dementia patients during the 12-month exercise intervention period. Additionally, the combined exercise program exhibited relatively high adherence to exercise (73% and 79%) for older participants (54) and dementia patients (53). Furthermore, balance training also indicated that adherence to exercise was relatively good, with 80% and 82% for aging people and stroke survivors, respectively (47, 55). Together, based on existing evidence, exercise is recommended as a practicable strategy for reducing fall risk for frail aging people and individuals with neurodegenerative disorders.

The heterogeneity across studies was high for falls and low for injurious falls and fractures. The high heterogeneity was mainly due to some participants experiencing high fall rates in some included studies (51, 56); for example, one study enrolled Parkinson’s disease people, and of the 195 who returned their falls calendars, 142 fell more than once and 86 people were multiple fallers who fell more than two times during follow-up (57). Twenty-one studies showed high risk and 16 studies marked unclear risk in at least one domain. Selective reporting was the most common domain that scored high risk, and the blinding domain was frequently ranked as an unclear risk in some studies. It is suggested that the risk of bias in studies on exercise and fall prevention was mainly caused by selective reporting and blinding. The sensitivity analysis demonstrated that our results on falls and fall-related injuries were stable, but not significant for fracture risk among frail aging people, which implied that the certainty of evidence on fracture prevention was not high. Although funnel plots for falls showed small asymmetry among neurodegenerative diseases, the evidence of the appearance of small-study effects was weak.

### The strength and limitations of this study

The strength of this meta-analysis was that based upon 64 RCTs, we had a unique opportunity to address the effects of exercise on falls and injuries among people with neurodegenerative disease or frail aging people. The findings have the potential to benefit those people through the provision of recommendations on exercise intervention protocols. The main limitation of this study was that the heterogeneity across studies for fall prevention was high, which might lower the certainty of evidence. Another limitation was that for exercise intervention studies, it was difficult to blind participants, so the included studies were frequently blinded for outcome assessment, which might cause a risk of bias. Additionally, the certainty of the evidence for fracture prevention in frail aging people was not high, which indicated that any explanations of the results should be taken with caution.

## Conclusion

This meta-analysis has provided pooled evidence to reveal that exercise is an effective strategy for preventing falls in frail aging people and individuals with neurological diseases. Moreover, exercise can also reduce fractures and fall-related injuries in aging people and prevent fall-induced injuries in patients with neurodegenerative diseases. However, the results should be interpreted cautiously due to the heterogeneity across studies, the single blinding strategy in exercise interventions, and the quality of some included studies. Nevertheless, our findings have clinical importance because it provides older people with high-risk conditions with an efficient strategy for the prevention of falls and subsequent injuries. Future studies should target the associations of exercise and fall prevention in people with other health conditions, e.g., hypertension, diabetes, or cardiovascular diseases.

## Data availability statement

The original contributions presented in the study are included in the article/**Supplementary Material**, further inquiries can be directed to the corresponding author.

## Author contributions

RZ and FF contributed to the study conception and drafted the manuscript. HX, YS, NL, XZ, XS, and XT contributed to the literature search, data extraction, data analysis, and data interpretation. All authors have given final approval to the version being published.

## References

[1] Montero-Odasso M van der Velde N Martin FC Petrovic M Tan MP Ryg J . World guidelines for falls prevention and management for older adults: a global initiative. Age Ageing (2022) 51(9):afac205. doi: 10.1093/ageing/afac205 36178003 PMC9523684

[2] Stewart Williams J Kowal P Hestekin H O'Driscoll T Peltzer K Yawson A . Prevalence, risk factors and disability associated with fall-related injury in older adults in low- and middle-incomecountries: results from the WHO study on global AGEing and adult health (SAGE). BMC Med (2015) 13:147. doi: 10.1186/s12916-015-0390-8 26099794 PMC4495610

[3] Haagsma JA Olij BF Majdan M van Beeck EF Vos T Castle CD . Falls in older aged adults in 22 European countries: incidence, mortality and burden of disease from 1990 to 2017. Inj Prev (2020) 26(Supp 1):i67–74. doi: 10.1136/injuryprev-2019-043347 PMC7571349 32111726

[4] Moreland B Kakara R Henry A . Trends in nonfatal falls and fall-related injuries among adults aged >/=65 years - United States, 2012-2018. MMWR Morb Mortal Wkly Rep (2020) 69(27):875–81. doi: 10.15585/mmwr.mm6927a5 PMC7732363 32644982

[5] Campbell AJ Borrie MJ Spears GF Jackson SL Brown JS Fitzgerald JL . Circumstances and consequences of falls experienced by a community population 70 years and over during a prospective study. Age Ageing (1990) 19(2):136–41. doi: 10.1093/ageing/19.2.136 2337010

[6] Tinetti ME Speechley M Ginter SF . Risk factors for falls among elderly persons living in the community. N Engl J Med (1988) 319(26):1701–7. doi: 10.1056/NEJM198812293192604 3205267

[7] Peel NM Kassulke DJ McClure RJ . Population based study of hospitalised fall related injuries in older people. Inj Prev (2002) 8(4):280–3. doi: 10.1136/ip.8.4.280 PMC1756575 12460962

[8] Burns ER Stevens JA Lee R . The direct costs of fatal and non-fatal falls among older adults — United States. J Saf Res (2016) 58:99–103. doi: 10.1016/j.jsr.2016.05.001 PMC6823838 27620939

[9] Al-Aama T . Falls in the elderly: spectrum and prevention. Can Fam Physician (2011) 57(7):771–6. PMC3135440 21753098

[10] Dionyssiotis Y . Analyzing the problem of falls among older people. Int J Gen Med (2012) 5:805–13. doi: 10.2147/IJGM.S32651 PMC3468115 23055770

[11] Pelicioni PHS Menant JC Latt MD Lord SR . Falls in Parkinson’s disease subtypes: risk factors, locations and circumstances. Int J Environ Res Public Health (2019) 16(12):2216. doi: 10.3390/ijerph16122216 31234571 PMC6616496

[12] Zhang W Low L-F Schwenk M Mills N Gwynn JD Clemson L . Review of gait, cognition, and fall risks with implications for fall prevention in older adults with dementia. Dementia Geriatric Cogn Disord (2019) 48(1-2):17–29. doi: 10.1159/000504340 31743907

[13] Goto Y Otaka Y Suzuki K Inoue S Kondo K Shimizu E . Incidence and circumstances of falls among community-dwelling ambulatory stroke survivors: a prospective study. Geriatrics Gerontol Int (2019) 19(3):240–4. doi: 10.1111/ggi.13594 30623545

[14] Fried LP Tangen CM Walston J Newman AB Hirsch C Gottdiener J . Frailty in older adults: evidence for a phenotype. J Gerontol A Biol Sci Med Sci (2001) 56(3):M146–56. doi: 10.1093/gerona/56.3.m146 11253156

[15] van den Bergh JP van Geel TA Geusens PP . Osteoporosis, frailty and fracture: implications for case finding and therapy. Nat Rev Rheumatol (2012) 8(3):163–72. doi: 10.1038/nrrheum.2011.217 22249162

[16] Bemben DA Fetters NL Bemben MG Nabavi N Koh ET . Musculoskeletal responses to high- and low-intensity resistance training in early postmenopausal women. Med Sci Sports Exerc (2000) 32(11):1949–57. doi: 10.1097/00005768-200011000-00020 11079527

[17] Patil R Uusi-Rasi K Tokola K Karinkanta S Kannus P Sievanen H . Effects of a multimodal exercise program on physical function, falls, and injuries in older women: a 2-year community-based, randomized controlled trial. J Am Geriatr Soc (2015) 63(7):1306–13. doi: 10.1111/jgs.13489 26115073

[18] Howe TE Rochester L Neil F Skelton DA Ballinger C . Exercise for improving balance in older people. Cochrane Database Syst Rev (2011) 11):CD004963. doi: 10.1002/14651858.CD004963.pub3 PMC11493176 22071817

[19] Beling J Roller M . Multifactorial intervention with balance training as a core component among fall-prone older adults. J Geriatr Phys Ther (2009) 32(3):125–33. doi: 10.1519/00139143-200932030-00008 20128337

[20] Emilio EJ Hita-Contreras F Jimenez-Lara PM Latorre-Roman P Martinez-Amat A . The association of flexibility, balance, and lumbar strength with balance ability: risk of falls in older adults. J Sports Sci Med (2014) 13(2):349–57. PMC3990889 24790489

[21] Aguero-Torres H Fratiglioni L Guo Z Viitanen M von Strauss E Winblad B . Dementia is the major cause of functional dependence in the elderly: 3-year follow-up data from a population-based study. Am J Public Health (1998) 88(10):1452–6. doi: 10.2105/AJPH.88.10.1452 PMC1508485 9772843

[22] Fasano A Canning CG Hausdorff JM Lord S Rochester L . Falls in Parkinson's disease: a complex and evolving picture. Mov Disord (2017) 32(11):1524–36. doi: 10.1002/mds.27195 29067726

[23] Kerse N Parag V Feigin VL McNaughton H Hackett ML Bennett DA . Falls after stroke: results from the Auckland regional community stroke (ARCOS) study, 2002 to 2003. Stroke (2008) 39(6):1890–3. doi: 10.1161/STROKEAHA.107.509885 18483413

[24] Caspersen CJ Powell KE Christenson GM . Physical activity, exercise, and physical fitness: definitions and distinctions for health-related research. Public Health Rep (1985) 100(2):126–31. PMC1424733 3920711

[25] Cadore EL Rodríguez-Mañas L Sinclair A Izquierdo M . Effects of different exercise interventions on risk of falls, gait ability, and balance in physically frail older adults: a systematic review. Rejuvenation Res (2013) 16(2):105–14. doi: 10.1089/rej.2012.1397 PMC3634155 23327448

[26] Zhao R Feng F Wang X . Exercise interventions and prevention of fall-related fractures in older people: a meta-analysis of randomized controlled trials. Int J Epidemiol (2017) 46(1):149–61. doi: 10.1093/ije/dyw142 27477031

[27] Zhao R Zhao M Xu Z . The effects of differing resistance training modes on the preservation of bone mineral density in postmenopausal women: a meta-analysis. Osteoporos Int (2015) 26(5):1605–18. doi: 10.1007/s00198-015-3034-0 25603795

[28] Karinkanta S Piirtola M Sievanen H Uusi-Rasi K Kannus P . Physical therapy approaches to reduce fall and fracture risk among older adults. Nat Rev Endocrinol (2010) 6(7):396–407. doi: 10.1038/nrendo.2010.70 20517287

[29] de Souto Barreto P Rolland Y Vellas B Maltais M . Association of long-term exercise training with risk of falls, fractures, hospitalizations, and mortality in older adults: a systematic review and meta-analysis. JAMA Intern Med (2018) 179(3):394–405. doi: 10.1001/jamainternmed.2018.5406 PMC6439708 30592475

[30] Sherrington C Fairhall N Wallbank G Tiedemann A Michaleff ZA Howard K . Exercise for preventing falls in older people living in the community: an abridged cochrane systematic review. Br J Sports Med (2020) 54(15):885–91. doi: 10.1136/bjsports-2019-101512 31792067

[31] Sherrington C Fairhall NJ Wallbank GK Tiedemann A Michaleff ZA Howard K . Exercise for preventing falls in older people living in the community. Cochrane Database Syst Rev (2019) 1:CD012424. doi: 10.1002/14651858.CD012424.pub2 30703272 PMC6360922

[32] Moher D Liberati A Tetzlaff J Altman DG PRISMA Group . Preferred reporting items for systematic reviews and meta-analyses: the PRISMA statement. Ann Intern Med (2009) 151(4):264–9. doi: 10.1371/journal.pmed.1000097 19622511

[33] Gillespie LD Robertson MC Gillespie WJ Sherrington C Gates S Clemson LM . Interventions for preventing falls in older people living in the community. Cochrane Database Syst Rev (2012) 9):CD007146. doi: 10.1002/14651858.CD007146.pub3 PMC8095069 22972103

[34] Higgins J Green S . Cochrane reviewers' handbook 5.0.1 (updated September 2008). The Cochrane Library, Chichester, UK: Wiley (2008).

[35] Martyn-St James M Carroll S . A meta-analysis of impact exercise on postmenopausal bone loss: the case for mixed loading exercise programmes. Br J Sports Med (2009) 43(12):898–908. doi: 10.1136/bjsm.2008.052704 18981037

[36] Zhao RQ Zhang MY Zhang Q . The effectiveness of combined exercise interventions for preventing postmenopausal bone loss: a systematic review and meta-analysis. J Orthop Sports Phys Ther (2017) 47(4):241–51. doi: 10.2519/jospt.2017.6969 28257620

[37] MeijersJMM HalfensRJG NeyensJCL LuikingYC VerlaanG ScholsJMGA . Predicting falls in elderly receiving home care: the role of malnutrition and impaired mobility. J nutrition Health Aging (2013) 16(7):654–8. doi: 10.1007/s12603-012-0010-7 22836709

[38] Stalenhoef PA Diederiks JPM de Witte LP Schiricke KH Crebolder HFJM . Impact of gait problems and falls on functioning in independent living persons of 55 years and over: a community survey. Patient Educ Couns (1999) 36(1):23–31. doi: 10.1016/s0738-3991(98)00071-8 10036557

[39] Baczkowicz D Szczegielniak J Proszkowiec M . Relations between postural stability, gait and falls in elderly persons–preliminary report. Ortop Traumatol Rehabil (2008) 10(5):478–85. 19043353

[40] El-Khoury F Cassou B Charles MA Dargent-Molina P . The effect of fall prevention exercise programmes on fall induced injuries in community dwelling older adults: systematic review and meta-analysis of randomised controlled trials. BMJ (2013) 347:f6234. doi: 10.1136/bmj.f6234 24169944 PMC3812467

[41] Zhao R Bu W Chen X . The efficacy and safety of exercise for prevention of fall-related injuries in older people with different health conditions, and differing intervention protocols: a meta-analysis of randomized controlled trials. BMC Geriatrics (2019) 19(1):341. doi: 10.1186/s12877-019-1359-9 31795944 PMC6892137

[42] Ashburn A Fazakarley L Ballinger C Pickering R McLellan LD Fitton C . A randomised controlled trial of a home based exercise programme to reduce the risk of falling among people with Parkinson's disease. J Neurol Neurosurg Psychiatry (2007) 78(7):678–84. doi: 10.1136/jnnp.2006.099333 PMC2117667 17119004

[43] Gao Q Leung A Yang Y Wei Q Guan M Jia C . Effects of tai chi on balance and fall prevention in Parkinson's disease: a randomized controlled trial. Clin Rehabil (2014) 28(8):748–53. doi: 10.1177/0269215514521044 24519923

[44] Brett L Stapley P Meedya S Traynor V . Effect of physical exercise on physical performance and fall incidents of individuals living with dementia in nursing homes: a randomized controlled trial. Physiother Theory Pract (2021) 37(1):38–51. doi: 10.1080/09593985.2019.1594470 30912690

[45] Dean CM Rissel C Sherrington C Sharkey M Cumming RG Lord SR . Exercise to enhance mobility and prevent falls after stroke: the community stroke club randomized trial. Neurorehabil Neural Repair (2012) 26(9):1046–57. doi: 10.1177/1545968312441711 22544817

[46] Marigold DS Eng JJ Dawson AS Inglis JT Harris JE Gylfadottir S . Exercise leads to faster postural reflexes, improved balance and mobility, and fewer falls in older persons with chronic stroke. J Am Geriatr Soc (2005) 53(3):416–23. doi: 10.1111/j.1532-5415.2005.53158.x PMC3226796 15743283

[47] Taylor-Piliae RE Hoke TM Hepworth JT Latt LD Najafi B Coull BM . Effect of tai chi on physical function, fall rates and quality of life among older stroke survivors. Arch Phys Med Rehabil (2014) 95(5):816–24. doi: 10.1016/j.apmr.2014.01.001 24440643

[48] Sparrow D DeAngelis TR Hendron K Thomas CA Saint-Hilaire M Ellis T . Highly challenging balance program reduces fall rate in Parkinson disease. J Neurol Phys Ther (2016) 40(1):24–30. doi: 10.1097/NPT.0000000000000111 26655100 PMC4681297

[49] El-Khoury F Cassou B Latouche A Aegerter P Charles MA Dargent-Molina P . Effectiveness of two year balance training programme on prevention of fall induced injuries in at risk women aged 75-85 living in community: ossebo randomised controlled trial. BMJ (2015) 351:h3830. doi: 10.1136/bmj.h3830 26201510 PMC4511529

[50] Buchner DM Cress ME de Lateur BJ Esselman PC Margherita AJ Price R . The effect of strength and endurance training on gait, balance, fall risk, and health services use in community-living older adults. J Gerontol A Biol Sci Med Sci (1997) 52(4):M218–24. doi: 10.1093/gerona/52A.4.M218 9224433

[51] Canning CG Sherrington C Lord SR Close JC Heritier S Heller GZ . Exercise for falls prevention in Parkinson disease: a randomized controlled trial. Neurology (2015) 84(3):304–12. doi: 10.1212/WNL.0000000000001155 PMC4335992 25552576

[52] Freiberger E Haberle L Spirduso WW Zijlstra GA . Long-term effects of three multicomponent exercise interventions on physical performance and fall-related psychological outcomes in community-dwelling older adults: a randomized controlled trial. J Am Geriatr Soc (2012) 60(3):437–46. doi: 10.1111/j.1532-5415.2011.03859.x 22324753

[53] Pitkala KH Poysti MM Laakkonen ML Tilvis RS Savikko N Kautiainen H . Effects of the Finnish Alzheimer disease exercise trial (FINALEX): a randomized controlled trial. JAMA Intern Med (2013) 173(10):894–901. doi: 10.1001/jamainternmed.2013.359 23589097

[54] Uusi-Rasi K Patil R Karinkanta S Kannus P Tokola K Lamberg-Allardt C . Exercise and vitamin d in fall prevention among older women: a randomized clinical trial. JAMA Intern Med (2015) 175(5):703–11. doi: 10.1001/jamainternmed.2015.0225 25799402

[55] Li F Harmer P Fisher KJ McAuley E Chaumeton N Eckstrom E . Tai chi and fall reductions in older adults: a randomized controlled trial. J Gerontol A Biol Sci Med Sci (2005) 60(2):187–94. doi: 10.1093/gerona/60.2.187 15814861

[56] Goodwin VA Richards SH Henley W Ewings P Taylor AH Campbell JL . An exercise intervention to prevent falls in people with Parkinson's disease: a pragmatic randomised controlled trial. J Neurol Neurosurg Psychiatry (2011) 82(11):1232–8. doi: 10.1136/jnnp-2011-300919 21856692

[57] Morris ME Taylor NF Watts JJ Evans A Horne M Kempster P . A home program of strength training, movement strategy training and education did not prevent falls in people with Parkinson’s disease: a randomised trial. J Physiother (2017) 63(2):94–100. doi: 10.1016/j.jphys.2017.02.015 28342682

