# Supplementary material for: Exercise for prevention of falls and fall-related injuries in neurodegenerative diseases and aging-related risk conditions: a meta-analysis
Source: Front Endocrinol (Lausanne). 2023 Jul 14;14:1187325. doi: 10.3389/fendo.2023.1187325 (PMC10393124; doi:10.3389/fendo.2023.1187325)
Supplement: Supplementary file 1 [file DataSheet_1.docx]

**Supplementary Files for**

**Exercise for prevention of falls and fall-related injuries in neurodegenerative diseases and aging-related risk conditions: a meta-analysis**

**Appendix:**

**Table S1.** Characteristics of included studies

**Table S2.** Risk of bias assessment

**Table S3.** Small-study effects examination

**Table S4.** Sensitivity analysis

**Figure S1.** Study selection

**Figure S2.** Funnel plots of falls for participants with neurodegenerative diseases

**Figure S3**. Funnel plots of fall-related injuries for participants with neurodegenerative diseases

**Figure S4.** Funnel plots of fractures for participants with neurodegenerative diseases

**Figure S5.** Funnel plots of falls for frail ageing people

**Figure S6**. Funnel plots of fall-related injuries for frail ageing people

**Figure S7**. Funnel plots of fractures for frail ageing people

**Text S1.** Search in PubMed

**Text S2.** Search in Cochrane Library

**Text S3.** Search in Search in SportDiscus

**Text S4.** Search in Web of Science

**Text S5.** Ongoing trials

**Text S6.** Included studies

| **Table S1.** Characteristics of included studies | | | | | | |
| --- | --- | --- | --- | --- | --- | --- |
| **Authors** | **Sample size (n); Study design; Follow-up** | **Ages (yrs)** | **Exercise intervention** | **CP** | **Control** | **Number of falls (exercise vs control)** |
| Arantes, 2015^[1]^, Brazil | 30 older people at high risk of falling; RCT; training: 3mos | 73.0 | Two times weekly of 1-h Balance exercises increased in difficulty  Settings: center based  Exercise types: balance | Sessions attended:22.1 | Neck/limb stretches and movements | 2, 5 |
| Ashburn, 2007^[2]^, UK | 142 Parkinson’s disease patients; RCT; training for 6 wks, following-up for 6 mos | 72.2 | Daily muscle strengthening, movement, balance training and walking, and encouraged  participants to continue their exercises after initial 6 wks  Settings: home based  Exercise types: combined protocols | 95.0% | Usual care | 46, 49 |
| Barnett, 2003^[3]^, Australia | 163 older people at high risk of falling; RCT; study duration: 1 yr | 74.9 | 1 h weekly of structured exercise, including balance, coordination, aerobic capacity and muscle strength training, plus home exercise  Settings: center/home based  Exercise types: combined protocols | 62.2% | Retained usual lifestyle | 27, 37 |
| Barreca, 2004^[4]^, Canada | 48 stroke survivors; RCT; 4-mo follow-up | 68.5 | 3 weekly extra exercises attaining standing from sitting from a variety of different surfaces  Settings: center/home based  Exercise types: combined protocols | NS | Conventional stroke rehabilitation | 3, 4 |
| Beling, 2009^[5]^, US | 23 older people at high risk of falling; RCT; exercise intervention:12 wks, following-up: 3 mos | 80.0 | Gravity exercises, balance training, and exercises to improve range of motion and strength, 3 times per wk  Settings: center based  Exercise types: combined protocols | NS | Maintain usual physical activity | 1, 4 |
| Beyer, 2007^[6]^, Denmark | 65 older people at high risk of falling; RCT; training duration: 6 mos, follow-up: 1yr | 78.1 | 60 mins of exercise programme, including flexibility, lower limb resistance exercise, and balance training, 2 times per wk  Settings: center based  Exercise types: combined protocols | 79% | Maintain  usual  physical  activity | 16, 20 |
| Boongird, 2017^[7]^,  Thailand | 439 older people at high risk of falling; RCT; study duration: 12 mos | 74.0 | Individual Otago Exercise Programme with lower extremities strengthening, stretching and balance training  Settings: home based  Exercise types: combined protocols | 57% | No intervention | 66, 88 |
| Brett, 2019^[8]^, Australia | 60 Dementia participants; RCT, ; study duration: 12 wks | 85.0 | Strength, balance and aerobic exercise lasting for 15 min, three times a week.  Settings: center based  Exercise types: combined protocols | 93% | Usual care | 0, 1 |
| Buchner, 1997^[9]^, US | 105 older people at high risk of falling  RCT; study duration:6 mos, follow-up: 18 mos | 75.0 | 1 h of endurance exercise, or trunk, upper and lower body strength training or combination of the two exercise types above, 3 times per wk  Settings: center/home based  Exercise types: combined protocols | 95% | Usual activity levels | 29, 18 |
| Canning, 2015^[10]^, Australia | 231 Parkinson’s disease patients; RCT; study duration 6 mos | 67.5 | 40 to 60 mins of progressive balance and lower limb strengthening exercises, 3 times a wk  Settings: center based  Exercise types: combined protocols | 72% | Usual care | 467, 810 |
| Cheng, 2001^[11]^,China | 54 stroke survivors; RCT; 6-mo follow-up | 62.7 | 30-min standing postural symmetry and repetitive sit-to-stand training, 5 days per wk, 3 wks  Settings: center based  Exercise types: balance exercise | NS | Conventional stroke rehabilitation | 5, 10 |
| Chivers Seymour, 2019^[12]^, UK | 474 Parkinson’s disease patients; RCT; training for 6 mons | 72.0 | Daily balance and strengthening exercises.  Settings: home based  Exercise types: combined protocols | NS | Usual care | 183, 187 |
| Clegg, 2014^[13]^, UK | 84 older people at high risk of falling; RCT; study duration: 12 wks | 79.7 | 3 times of 10-15 repetitions of strengthening exercises for the muscle groups required for basic mobility skills, 5 days per wk  Settings: home based  Exercise types: strength training | 64.0% | Receive usual care | 7, 8 |
| Clemson. 2010^[14]^, Australia | 34 older people at high risk of falling; RCT; study duration: 6 mos | 81.5 | progressive balance and strength training embedded in daily life activities  Settings: home based  Exercise types: combined protocols | NS | No exercise intervention | 12, 35 |
| Clemson, 2012^[15]^, Australia | 317 older people at high risk of falling; RCT; study duration: 12 mos | 83.4 | LiFE approach: exercises of balance and strength training and integrated selected activities into everyday routines; or structured programme: exercises for balance and lower limb strength, 3 times a wk  Settings: home based  Exercise types: combined protocols | NS | Gentle and flexibility exercises | 365, 224 |
| Dadgari, 2016^[16]^, Iran | 451 older people at high risk of falling; RCT; study duration: 6 mos | 70.3 | 40-45 mins of strength and balance retraining, 3 times per week  Settings: home based  Exercise types: combined protocols | NS | Health education | 202, 258 |
| de Souto Barreto, 2017^[17]^, France | 97 participants with Dementia; RCT; study duration: 6 mos | 87.6 | A combination of balance, aerobic, and strength training exercises, twice per week for 60 mins.  Settings: center based  Exercise types: combined protocols | 74% | Usual  care | 25, 36 |
| El-Khoury, 2015^[18]^, France | 706 older people at high risk of falling; RCT; study duration: 2 yrs | 79.7 | Weekly supervised progressive balance training, supplemented by six weekly individually prescribed home exercises  Settings: center based  Exercise types: balance | NS | No exercise intervention | 533, 640 |
| Elley, 2008^[19]^, New Zealand | 312 older people at high risk of falling; RCT; study duration: 1 yr | 80.8 | Daily strength and balance exercise  Settings: home based  Exercise types: combined protocols | NS | Usual  care and social visits | 285, 299 |
| Freiberger, 2012^[20]^, Germany | 280 older people at high risk of falling; RCT; training for 12 mos and following-up for 24 mons | 76.1 | 1 h of strength and balance training, or plus progressive strength training and challenging balance exercises, or plus endurance training, 2 times per wk  Settings: center based  Exercise types: combined protocols | 84% | No exercise intervention | 125, 82 |
| Gao, 2014^[21]^, China | 76 Parkinson’s disease patients; RCT; training for 12 wks and following-up for 6 mons | 68.9 | 60 mins of Yang Style Tai Chi exercise, 3 times per wk  Settings: center based  Exercise types: balance | 86.0% | No exercise intervention | 8, 19 |
| Goodwin, 2011^[22]^, UK | 130; RCT; 10-wk training and 5-mon follow-up | 71.1 | Weekly strength and balance training programme with home exercises  Settings: home based  Exercise types: combined protocols | NS | No exercise intervention | 1507, 3981 |
| Haines, 2009^[23]^, Australia | 53 older people at high risk of falling; RCT; study duration: 6 mos | 80.7 | A progressive exercise programme combining lower limb strength and balance exercises  Settings: home based  Exercise types: combined protocols | NS | Usual care | 21, 51 |
| Hauer, 2001^[24]^, Germany | 57 older people at high risk of falling; RCT; exercise intervention for 3 mos, follow-up for 6 mos | 82.2 | 90 mins of lower-extremity progressive resistance training and progressive functional  balance training, 3 times per wk  Settings: center based  Exercise types: combined protocols | 85.0% | Motor placebo activities | 10, 13 |
| Hirase, 2015^[25]^, Japan | 93 older people at high risk of falling; RCT; study duration: 4 mos | 82.1 | once weekly of 60 mins balance training performed either on foam rubble or stable surface, followed a home-based exercise routine  Settings: center based  Exercise types: balance | 95.5%, 93.3% | Weekly  social visit | 26, 27 |
| Iliffe, 2014^[26]^, UK | 1256 older people at high risk of falling; RCT; training for 6 mos, following-up for 1 yr | 72.9 | Centre-based exercise: 1 h of upper body and lower body strength training, plus 2 times of walking per wk; home-based exercise: 3 times per wk of muscle strengthening and balance exercises plus 2 times of walking  Settings: center/home based  Exercise types: combined protocols | NS | No exercise intervention | 402, 271 |
| Kim, 2014^[27]^, Japan | 105 older people at high risk of falling; RCT; Training for 3 mos and following-up for 1 yr | 78.1 | 60 mins of muscle strength and balance training, 2 times per wk  Settings: center based  Exercise types: combined protocols | 75.3% | No exercise intervention | 10, 21 |
| Kovacs, 2013^[28]^, Hungary | 86 older people at high risk of falling; RCT; study duration: 1 yr | 77.8 | Progressive exercise program including strengthening and balance exercise, supplemented with a 30-min walking training, 2 times a wk  Settings: center/home based  Exercise types: combined protocols | 78.0% | No exercise intervention | 22, 29 |
| Kruse, 2010^[29]^, US | 79 older people at high risk of falling; RCT; study duration: 1 yr | 65.6 | 8 individual sessions of exercises to progressively strengthen legs and promote balance, and 3 additional weekly 1-hour sessions at home; followed by continuing their leg strengthening and balance exercises in home  Settings: center/home based  Exercise types: combined protocols | 45.0% | Received diabetes self-care instruction | 33, 31 |
| Latham, 2003^[30]^, Australia | 243 older people at high risk of falling; RCT; training for 10 wks, following-up for 6 mos | 79.0 | 3 sets of 8 repetitions of resistance exercise consisted of a quadriceps exercise program using adjustable ankle cuff weights undertaken, 3 times per wk  Settings: home based  Exercise types: resistance exercise | 82% | No exercise intervention | 164, 149 |
| Li, 2012^[31]^, Portland | 195 Parkinson’s Disease patients; RCT; study duration: 6 mos | 64.0 | 60-min Tan Chi Quan exercise, twice weekly  Settings: center based  Exercise types: balance exercise | 77% | Usual care | 62, 186 |
| Lin, 2007^[32]^, China | 150 older people at high risk of falling; RCT; training for 4 wks, following-up for 6 mos | 76.8 | 50 mins of individualized stretching, muscle strengthening, and balance training  Settings: home based  Exercise types: combined protocols | NS | Education on fall prevention | 1, 2 |
| Liu-Ambrose, 2008^[33]^, Australia | 74 older people at high risk of falling; RCT; study duration: 1 yr | 82.3 | 30 mins of home-based strength and balance-retraining programme, 3 times per wk, plus at least twice weekly walking  Settings: home based  Exercise types: combined protocols | 57.0% | No exercise intervention | 12, 16 |
| Liu-Ambrose, 2019^[34]^, Canada | 345 older people at high risk of falling; RCT, study duration: 12 mos | 81.6 | 3 times weekly of Otago Exercise promoting balance and strength retraining and at least 30 mins of walking  Settings: home based  Exercise types: combined protocols | 63% | Usual care | 204, 235 |
| Logghe, 2009^[35]^, Netherlands | 269 older people at high risk of falling; RCT; training for 13 wks, following-up for 1 yr | 77.3 | 1 h of Taichi exercise, 2 times per wk  Settings: center based  Exercise types: Balance exercise | 47.0% | No exercise intervention | 115, 90 |
| Luukinen, 2007^[36]^, Finland | 484 older people at high risk of falling; RCT; study duration: 16 mos | 88.0 | 5-15 repetitions of daily home exercise or group exercise, including walking exercises or self-care exercises  Settings: center/home based  Exercise types: Aerobic exercise | NS | No exercise intervention | 126, 136 |
| Mahoney, 2007^[37]^, US | 349 older people at high risk of falling; RCT; study duration: 1 yr | 80.0 | 4 to 5 days weekly walking and 2 to 3  days weekly standing balance exercises  Settings: center based  Exercise types: combined protocols | NS | No exercise intervention | 327, 404 |
| Marigold, 2005^[38]^, Canada | 61 stroke survivors; RCT; 10-wk intervention and 1-yr follow-up | 78.5 | challenged dynamic balance, and the tasks progressively increased in difficulty  Settings: center based  Exercise types: Balance exercise | 93% | Stretching exercise | 25, 75 |
| Morgan, 2004^[39]^, US | 294; RCT; training for 8 wks, following-up for 1 yr | 80.6 | 45 mins of low-intensity group exercises to improve muscle strength, joint flexibility, balance and gait, 3 times per wk  Settings: center based  Exercise types: combined protocols | 82.9% | Continue their usual activities | 34, 34 |
| Morris, 2015^[40]^, Australia | 210 Parkinson’s disease patients; RCT; training for 8 wks, following-up for 1 yr | 67.7 | Once weekly progressive functional resistance exercises, plus 1 home exercise program  Settings: center/home based  Exercise types: resistance exercise | 90.0% | Conducted the life-skill sessions | 193, 913 |
| Nitz, 2004^[41]^, Australia | 73 older people at high risk of falling; RCT; training for 10 wks, following-up for 3 mos | 75.8 | 1 h of weekly balance training in small groups using workstation format  Settings: center based  Exercise types: Balance exercise | NS | No exercise intervention | 21, 22 |
| Nyman, 2019^[42]^, UK | 85 Dementia patients; RCT; study duration: 6 mos | 78.1 | Weekly Tai Qi Quan exercise plus home practice.  Settings: center/home based  Exercise types: Balance exercise | 55% | Usual care | 61, 95 |
| Ohman, 2016^[43]^, Finland | 194 Dementia patients; RCT; 12-mo duration | 67.1 | 2 weekly 1-h exercise to improve everyday skills, including weight lifting, walking, balance, *etc*.  Settings: home based  Exercise types: combined protocols | NS | Usual care | 203, 203 |
| Pang, 2018^[44]^, China | 84 stroke survivors; RCT; 8-wk training and 6-mon follow-up | 78.2 | 3 weekly 60-mins of balance and dynamic mobility training programme  Settings: center based  Exercise types: Balance exercise | 93 | Sham exercise | 4, 20 |
| Patil 2015^[45]^, Finland | 409 older people at high risk of falling; RCT; training duration: 1 yr; following-up: 1 yr | 74.2 | Twice weekly balance challenging, weight bearing, strengthening, agility, and functional exercises for the first 12 mos, and once weekly group and home exercise for remaining 12 mos  Settings: center/home based  Exercise types: combined protocols | 73% & 66% | Maintained routine physical activity | 448, 480 |
| Pitkala, 2013^[46]^, Finland | 210 Dementia patients; RCT; study duration: 1 yr | 68.9 | 1 h of endurance, balance, and strength training, 2 times per wk  Settings: center/home based  Exercise types: combined protocols | 78.6% | Usual care | 101, 171 |
| Puente-González, 2021^[47]^, Spain | 72 Dementia patients; RCT; study duration: 6 mos | 71.6 | 45-50 mins of balance, aerobic strength training exercises, Three times weekly  Settings: center based  Exercise types: combined protocols | NS | Usual care | 11, 11 |
| Rolland, 2007^[48]^, France | 134 ambulatory patients with Dementia; RCT; study duration: 1 yr | 83.0 | 1 h, twice weekly of walk, strength, balance, and flexibility training  Settings: center based  Exercise types: combined protocols | 33.2% | Usual care | 139, 136 |
| Rosendahl, 2008^[49]^, Sweden | 191 older people; RCT; training for 3 mos, following-up for 6 mos | 84.8 | Tai Chi movements for balance and leg strength, instructed to use daily for 20 mins or get involved in community exercise programme for 45 mins 3 times per wk  Settings: center/home based  Exercise types: combined protocols | 76.0% | No exercise intervention | 141, 200 |
| Sakamoto, 2006^[50]^, Japan | 553 older people at high risk of falling; RCT; study duration: 6 mos | 81.6 | 1 min of one-leg standing with eyes open, 3 times a day  Settings: home based  Exercise types: balance exercise | NS | No exercise intervention | 118, 121 |
| Sakamoto, 2013^[51]^, Japan | 1365 older people at high risk of falling; RCT; study duration: 6 mos | 75.0 | 1 min of right leg standing with eyes open, and then their left leg for another min, for a total of 2 mins, 3 times a day  Settings: home based  Exercise types: balance exercise | NS | No exercise intervention | 114, 152 |
| Salminen, 2009^[52]^, Finland | 591 older people at high risk of falling; RCT; study duration: 1 yr | 65.0 | 15 mins of balance, coordination and weight-shifting exercises, 20 mins of circuit training, and muscle strength training, 3 times per wk at home  Settings: center/home based  Exercise types: combined protocols | 58.0% | No exercise intervention | 243, 271 |
| Sherrington, 2014^[53]^, Australia | 340; RCT; study duration: 1 yr | 81.2 | 20 to 30 mins program of lower limb balance and strengthening exercises, 6 times per wk  Settings: combined based  Exercise types: combined protocols | 60% | Usual care | 177, 123 |
| Siegrist, 2016^[54]^, Germany | 378 older people at high risk of falling; RCT; training duration: 12 wks; follow-up: 12 mos | 78.0 | 1 h weekly of muscle strengthening and challenging balance training exercises, combined with a 12 wk home-based exercise program  Settings: center/home based  Exercise types: combined protocols | 82% | No exercise intervention | 291, 367 |
| Skelton, 2005^[55]^, UK | 100 older people at high risk of falling; RCT; study duration: 36 wks | 73.0 | 1 h weekly of challenging balance exercises, plus 30 mins twice welly home exercise  Settings: center/home based  Exercise types: balance exercise | NS | Sham exercise | 391, 352 |
| Smulders, 2010^[56]^, Netherlands | 96 older people at high risk of falling; RCT; training for 3 mos, following-up for 1 yr | 71.1 | Weight-bearing and walking exercises, and gait correction and fall prevention training programmes  Settings: center based  Exercise types: combined protocols | 92.8% | Usual lifestyle | 34, 52 |
| Sparrow, 2016^[57]^, US | 23 Parkinson’s Disease patients; RCT; training for 3 mos | 66.7 | 90 mins of balancer and strength training exercises, twice weekly  Settings: center based  Exercise types: combined protocols | NS | Usual care | 25, 25 |
| Steadman, 2003^[58]^, UK | 198 older people at high risk of falling; RCT; training for 6 wks, following-up for 24 wks | 82.7 | 45 mins of enhanced balance training in addition to conventional physiotherapy, 2 times per wk  Settings: center based  Exercise types: balance exercise | NS | Conventional physiotherapy | 41, 38 |
| Taylor-Piliae, 2014^[59]^, US | RCT stroke survivors; study duration: 12 wks | 79.8 | 1 h of Taichi exercise, 3 times per wk  Settings: center based  Exercise types: balance exercise | 82.0% | Usual care | 16, 28 |
| Toots, 2018^[60]^, Sweden | 196 Alzheimer’s disease; RCT; training 4 mons and following-up for 12 mons | 85.2 | 2-3 times weekly of high-intensity functional exercise  Settings: home based  Exercise types: combined protocols | 73% | Seated attention | 232, 241 |
| Uusi-Rasi, 2015^[61]^, Finland | 409 older people at high risk of falling; RCT; study duration: 2 yrs | 74.3 | Balance challenging, weight bearing, strengthening, agility, and functional exercises, plus 5-15 mins of home-training program, 2 times a wk for the first 12 months and once a wk for the remaining 12 months  Settings: center based  Exercise types: combined protocols | 72.8% | No exercise intervention | 121, 118 |
| Wesson, 2013^[62]^, Australia | 22 Dementia patients; RCT; study duration: 12 wks | 61.2 | Individually tailored strength and balance exercises, 3 times per wk  Settings: home based  Exercise types: combined protocols | 72.7% | Usual care | 5, 11 |
| Wolf, 1996^[63]^, US | 200 osteoporotic people; RCT; training for 15 wks, following-up for 8 mos | 65.0 | Twice weekly Tai Chi Quan exercise  Settings: center based  Exercise types: balance exercise | NS | No exercise intervention | 127, 170 |
| Yang,2012^[64]^, Australia | 121 older people at high risk of falling; RCT; study duration: 24 wks | 81.0 | 20 mins of strength and balance training, plus 30-min waling, 5 times per wk  Settings: home based  Exercise types: combined protocols | NS | No exercise intervention | 14, 26 |

**Notes**: *yr(s):* year(s); *mo(s)*: month(s); *wk(s)*: week(s); *h(s):* hour(s); *min(s)*: minute(s); *RM*: repetition; *NS*: no statement; *reps:* repetitions; *CP:* compliance

**Table S2.** Risk of bias assessment

| **Author** | **Sequence Generation** | **Allocation Concealment** | **Blinding** | **Incomplete Outcome Data** | **Selective Reporting** |
| --- | --- | --- | --- | --- | --- |
| Arantes, 2015 | unclear | unclear | unclear | unclear | high |
| Ashburn, 2007 | low | low | low | low | low |
| Barnett, 2003 | unclear | low | low | low | low |
| Barreca, 2004 | low | unclear | unclear | low | high |
| Beling, 2009 | unclear | unclear | unclear | high | high |
| Beyer, 2007 | low | unclear | unclear | low | high |
| Boongird, 2017 | low | low | low | low | low |
| Brett, 2019 | low | low | low | low | unclear |
| Buchner, 1997 | low | unclear | low | low | low |
| Canning, 2015 | low | low | low | low | low |
| Cheng, 2001 | low | unclear | unclear | low | high |
| Chivers Seymour, 2019 | low | low | low | low | low |
| Clegg, 2014 | low | low | low | low | low |
| Clemson, 2010 | low | low | low | unclear | unclear |
| Clemson, 2012 | low | low | low | low | low |
| Dadgari, 2016 | low | unclear | unclear | unclear | unclear |
| de Souto Barreto, 2017 | low | low | low | low | low |
| El-Khoury, 2015 | low | low | low | low | low |
| Elley, 2008 | low | low | low | low | low |
| Freiberger, 2012 | low | low | unclear | low | high |
| Gao, 2014 | low | unclear | low | low | unclear |
| Goodwin, 2011 | low | low | low | low | low |
| Haines, 2009 | low | low | low | low | low |
| Hauer, 2001 | unclear | unclear | low | low | low |
| Hirase, 2015 | unclear | low | unclear | low | high |
| Iliffe, 2014 | low | low | high | low | low |
| Kim, 2013 | low | low | low | low | low |
| Kovacs, 2013 | low | low | low | low | low |
| Kruse, 2010 | low | low | low | low | high |
| Latham, 2003 | low | low | low | low | low |
| Li, 2012 | low | low | low | low | low |
| Lin, 2007 | low | unclear | unclear | unclear | high |
| Liu-Ambrose, 2008 | low | low | low | low | low |
| Liu-Ambrose, 2018 | low | low | low | low | low |
| Logghe, 2009 | low | low | low | low | low |
| Luukinen, 2007 | low | unclear | low | low | low |
| Mahoney, 2007 | low | unclear | low | low | low |
| Marigold, 2005 | low | low | low | low | low |
| Morgan, 2004 | unclear | unclear | unclear | low | high |
| Morris, 2015 | low | low | low | low | low |
| Nitz, 2004 | low | unclear | low | high | high |
| Nyman, 2019 | low | low | low | low | low |
| Ohman, 2016(a) | low | low | unclear | low | low |
| Ohman, 2016(b) | low | low | unclear | low | low |
| Pang, 2018 | low | low | low | low | low |
| Patil, 2015 | unclear | unclear | unclear | low | low |
| Pitkala, 2013 | low | low | low | low | low |
| Puente-González, 2021 | high | high | low | low | low |
| Rolland, 2007 | low | low | low | low | high |
| Rosendahl, 2008 | low | low | low | low | low |
| Sakamoto, 2006 | unclear | unclear | unclear | high | high |
| Sakamoto, 2013 | low | low | unclear | low | unclear |
| Salminen, 2009 | low | low | low | low | low |
| Sherrington, 2014 | high | low | low | low | low |
| Siegrist, 2016 | low | unclear | low | low | low |
| Skelton, 2005 | low | unclear | low | unclear | low |
| Smulders, 2010 | low | unclear | low | low | low |
| Sparrow, 2016 | low | low | low | low | high |
| Steadman, 2003 | low | unclear | low | high | high |
| Taylor-Piliae, 2014 | low | low | low | low | low |
| Toots, 2018 | low | low | unclear | low | high |
| Uusi-Rasi, 2015 | low | low | low | low | low |
| Wesson, 2013 | low | low | low | low | high |
| Wolf, 2003 | unclear | unclear | low | low | unclear |
| Yang, 2012 | low | low | low | low | high |

**Notes:** Blind for outcome assessment.

**Table S3.** Egger's test for small-study effects

|  | **Falls** | **Fracture** | **Fall-related Injuries** |
| --- | --- | --- | --- |
| Neurodegenerative Diseases (p value) | 0.068 | 0.151 | 0.058 |
| Frail Aging People  (p value) | 0.888 | 0.068 | 0.059 |

**Table S4.** Sensitivity analysis

|  | **Studies (n)** | **Falls** | **Studies (n)** | **Fracture** | **Studies (n)** | **Fall-related Injuries** |
| --- | --- | --- | --- | --- | --- | --- |
| Neurodegenerative Diseases | 7 | 0.67 (0.48–0.93) | 4 | 1.33 (0.70–2.52) | 6 | 0.71 (0.51–0.98) |
| Frail Aging People | 15 | 0.85 (0.78–0.93) | 3 | 0.71 (0.27–1.91) | 6 | 0.91 (0.84–0.98) |

**Notes:** Sensitivity analysis was performed after dropping studies with either high risk or unclear.

**Figure S1**

**
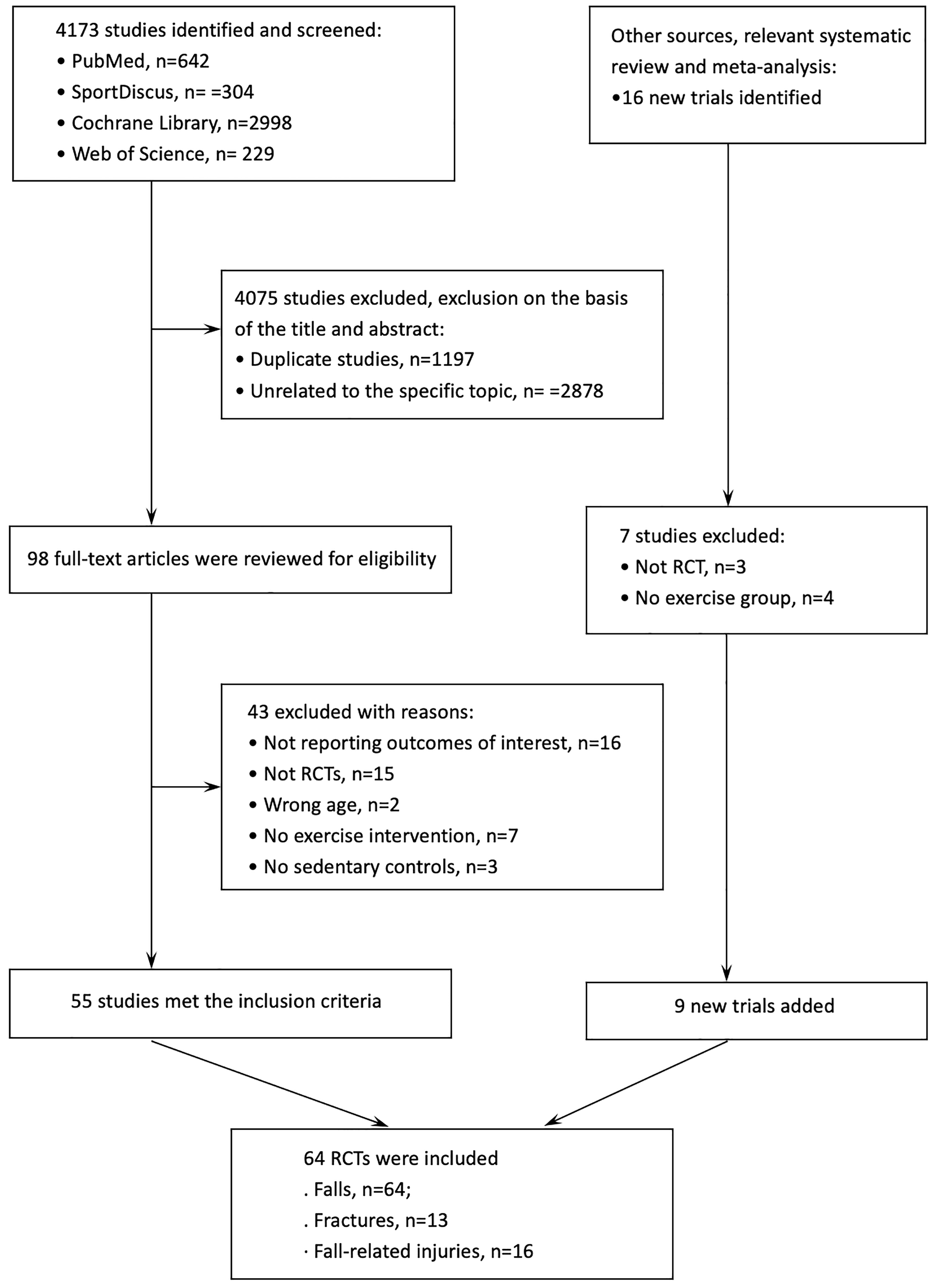
**

**Figure S2**


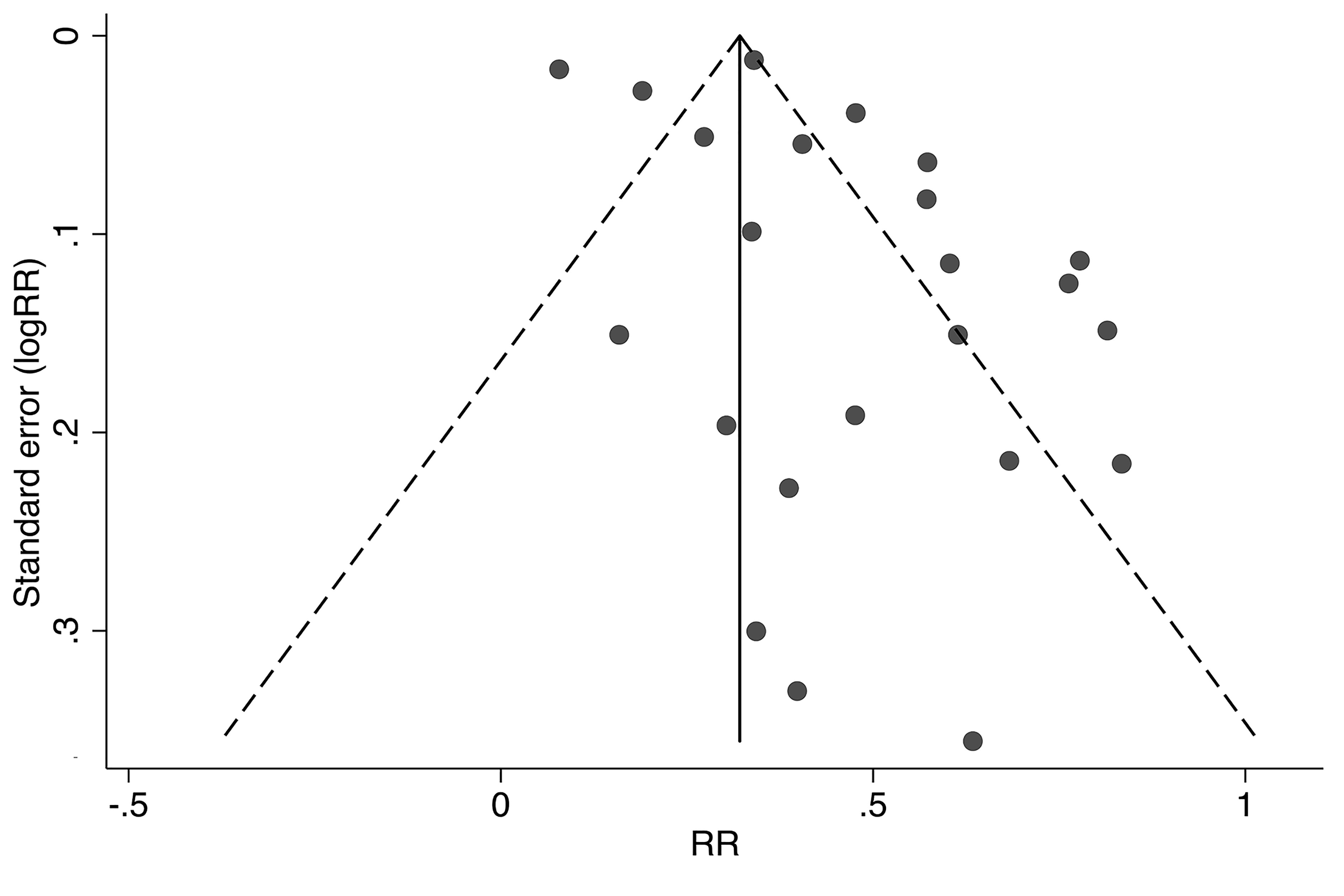


**Figure S3**


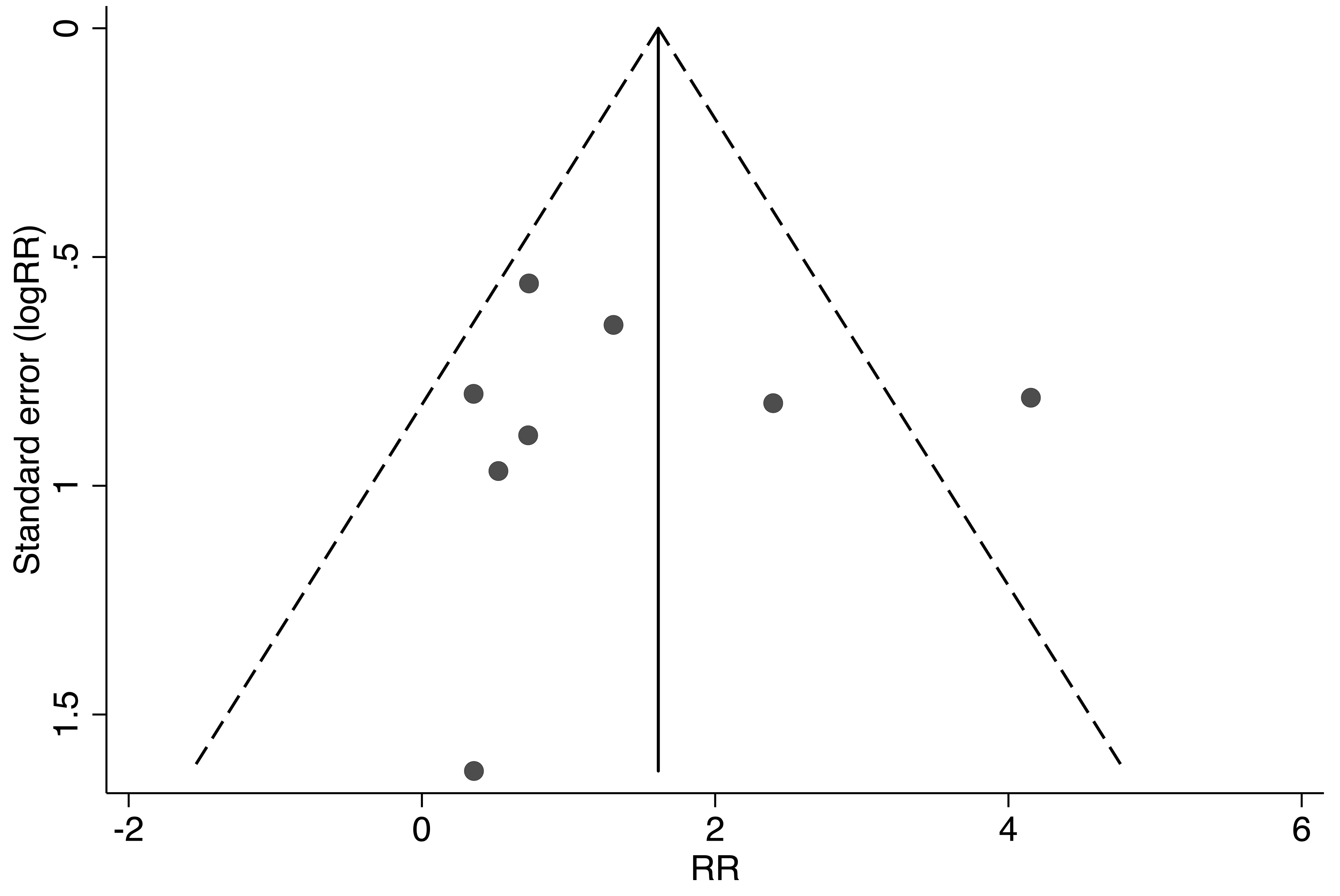


**Figure S4**


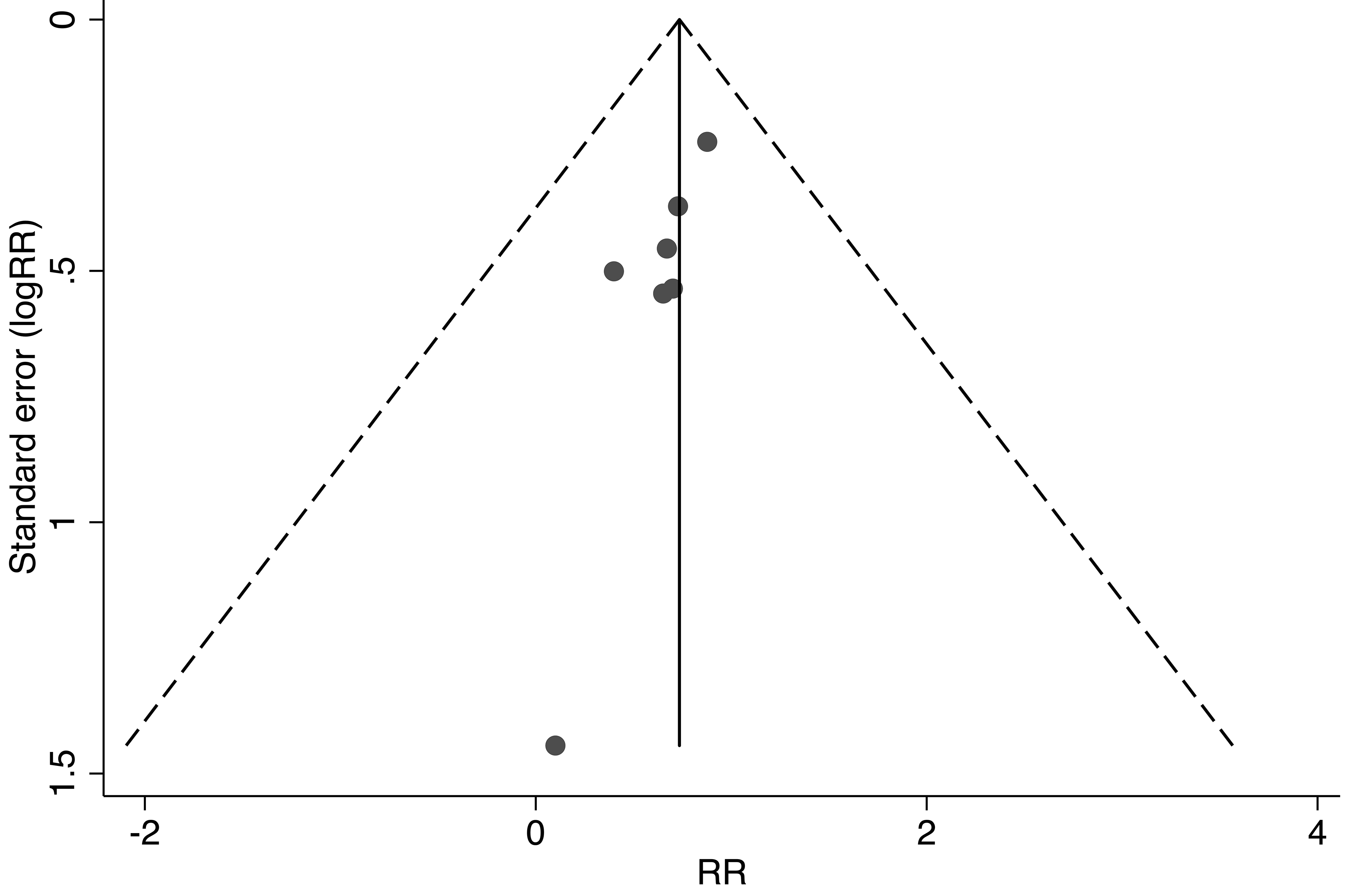


**Figure S5**


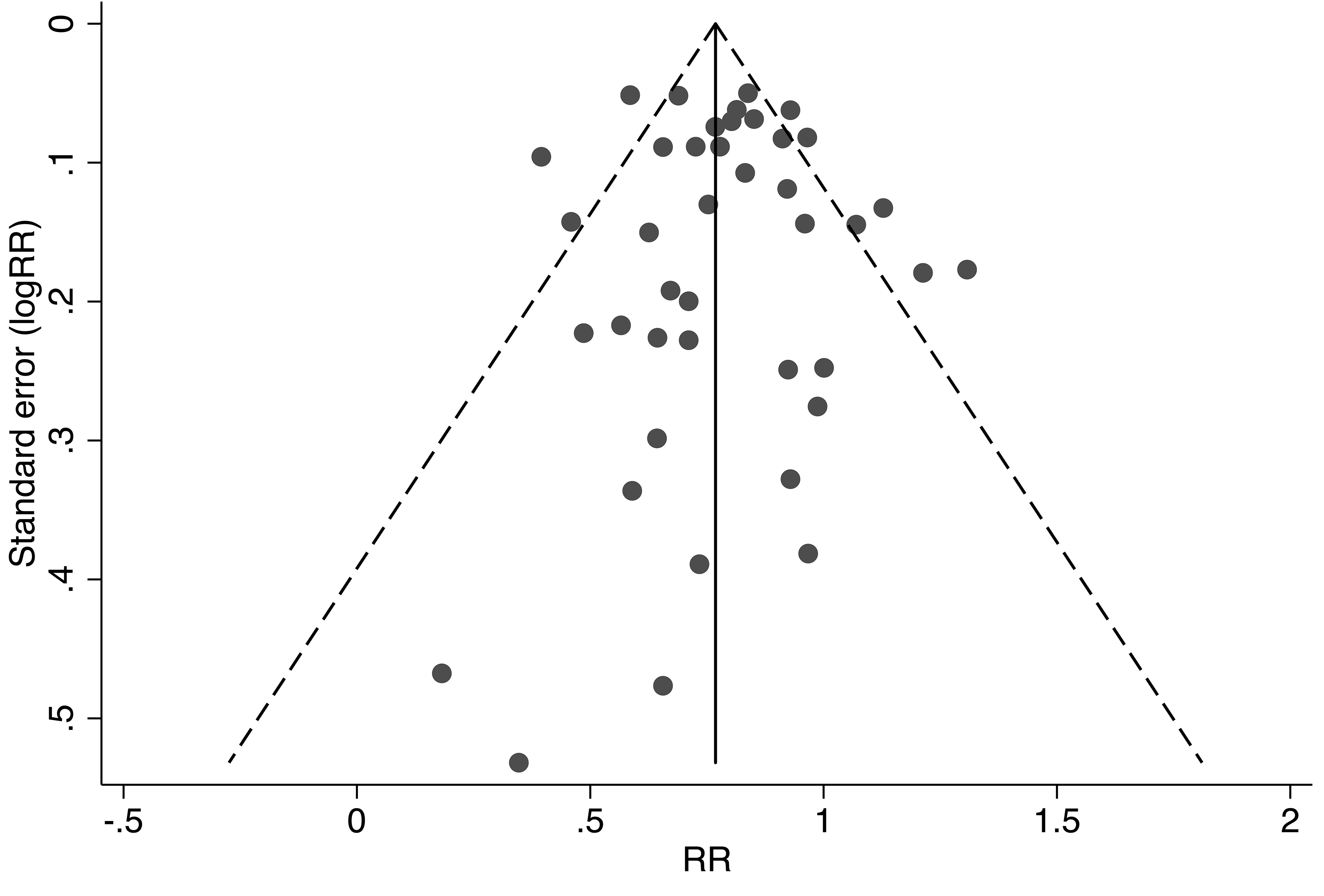


**Figure S6**


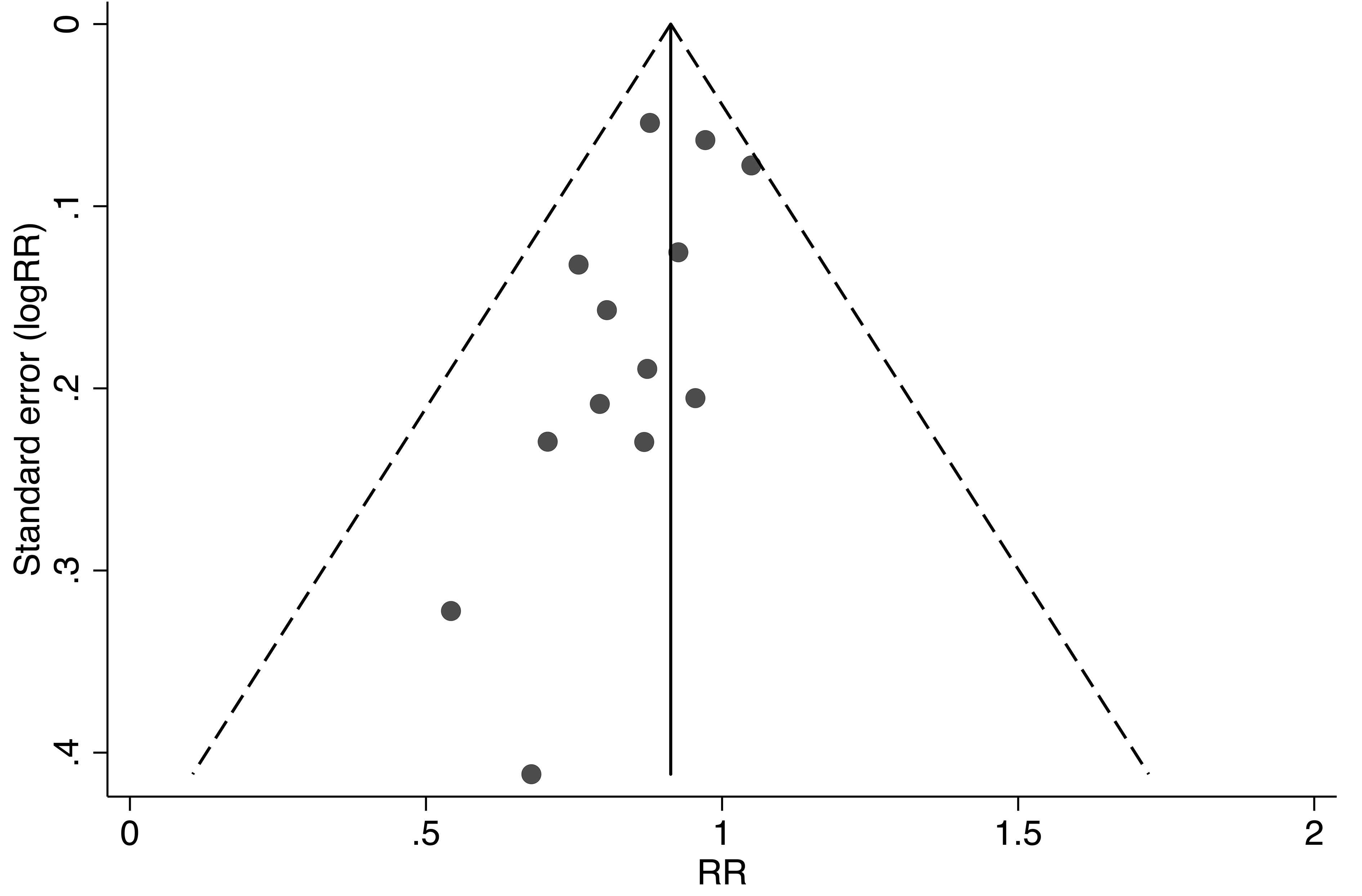


**Figure S7**


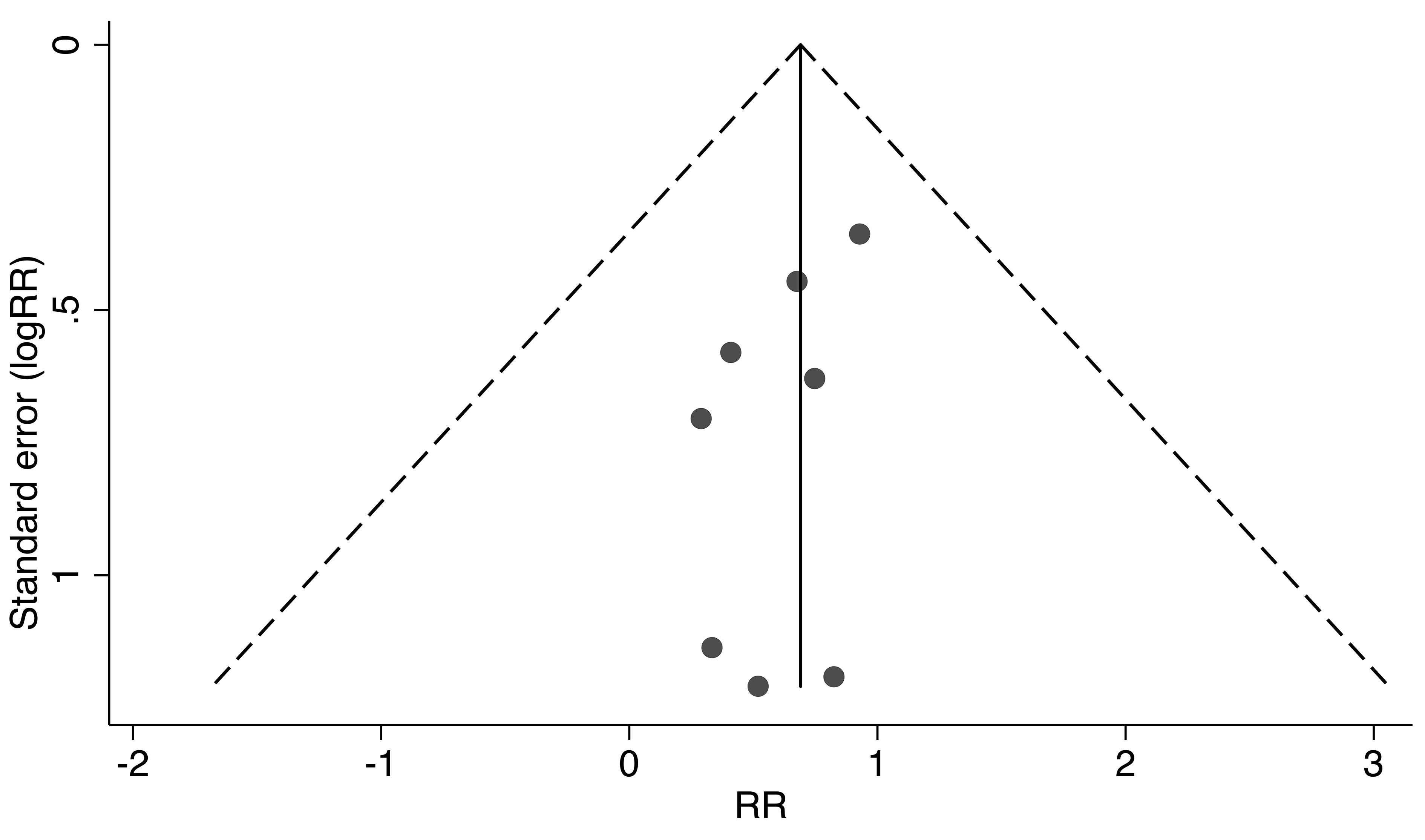


**Text S1.** **Search in PubMed**

Date Run: 1/1/2023

Number of studies retrieved: 642

search query

1 exercis*[Title/Abstract]

2 physical activit*[Title/Abstract]

3 resistance training[Title/Abstract]

4 strength training[Title/Abstract]

5 balance training[Title/Abstract]

6 walking[Title/Abstract]

7 running[Title/Abstract]

8 aerobic program*[Title/Abstract]

9 physical therapy[Title/Abstract]

10 physical rehabilitation[Title/Abstract]

11 (1 OR 2 OR 3 OR 4 OR 5 OR 6 OR 7 OR 8 OR 9 OR 10)

12 (fall* [Title/Abstract] OR fractur* [Title/Abstract])

13 (dementia[Title/Abstract] OR Alzheimer’s disease[Title/Abstract])

14 stroke[Title/Abstract]

15 Parkinson’s diseases[Title/Abstract]

16 (high risk[Title/Abstract] OR high* risk[Title/Abstract] OR high-risk[Title/Abstract])

17 (12 OR 13 OR 14 OR 15 OR 16)

18 (11 AND 17)

19 randomized controlled trial*[Title/Abstract]

20 (18 AND 19)

21 older adult*[Title/Abstract]

22 (older population[Title/Abstract] OR older people[Title/Abstract])

23 elder*[Title/Abstract]

24 (21 OR 22 OR 23)

25 (20 AND 24)

**Text S2: Search in Cochrane Library**

Date Run: 1/1/2023

Number of studies retrieved: 2998

**Id Search**

#1 (exercis*):ti,ab,kw

#2 (physical activit*):ti,ab,kw

#3 (resistance training):ti,ab,kw

#4 (strength training):ti,ab,kw

#5 (balance training):ti,ab,kw

#6 (walking):ti,ab,kw

#7 (running):ti,ab,kw

#8 (aerobic program*):ti,ab,kw

#9 (physical therapy):ti,ab,kw

#10 (physical rehabilitation):ti,ab,kw

#11 #1 OR #2 OR #3 OR #4 OR #5 OR #6 OR #7 OR #8 OR #9 OR #10

#12 (fall* OR fractur*):ti,ab,kw

#13 (dementia OR Alzheimer’s disease):ti,ab,kw

#14 (stroke):ti,ab,kw

#15 (Parkinson’s disease):ti,ab,kw

#16 (high risk OR high* risk OR high-risk):ti,ab,kw

#17 #12 OR #13 OR #14 OR #15 OR #16

#18 #11 AND #17

#19 (randomized controlled trial*):ti,ab,kw

#20 #18 AND #19

#21 (older adult*):ti,ab,kw

#22 (older population OR older people):ti,ab,kw

#23 (elder*):ti,ab,kw

#24 #21 OR #22 OR #23

#25 #20 AND #24

**Text S3: Search in SportDiscus**

Date Run: 1/1/2023

Number of studies retrieved: 304

| S1 | TI exercis* OR TI physical activit* OR TI resistance training OR TI strength training OR TI balance training OR TI walking OR TI running OR TI aerobic program OR TI physical therapy OR TI physical rehabilitation |
| --- | --- |
| S2 | TI older adult* OR TI older population OR TI older people OR TI elder* OR AB old* |
| S3 | TI fall* OR fractur* |
| S4 | TI dementia OR TI stroke OR TI Parkinson’s disease OR TI high risk |
| S5 | AB randomized controlled trial* OR AB clinical trial OR AB random* |
| S6 | S1 AND S2 AND S3 AND S4 AND S5 |

**Text S4: Search in Web of Science**

Date Run: 1/1/2023

Number of studies retrieved: 229

| **Set** | **Search** |
| --- | --- |
| # 1 | TS=(exercis* OR physical activit* OR resistance training OR strength training OR balance training OR walking OR running OR aerobic program OR physical therapy OR physical rehabilitation) |
| # 2 | TS=(fall* OR fractur*) |
| # 3 | TS =( dementia OR stroke OR Parkinson’s disease OR high risk) |
| # 4 | TI=(randomized controlled trial* OR randomised controlled trial*) |
| # 5 | TS=(older adult* OR older population OR older people OR elder*) |
| # 6 | #1 AND #2 AND #3 AND #4 AND #5 |

**Text S5. Ongoing trials:**

**A. the WHO International Clinical Trials Registry Platform (ICTRP)**

| No. | Recruitment status |  | Main ID | Public Title | Date of Registration | Results available |
| --- | --- | --- | --- | --- | --- | --- |
| 1 | Recruiting | No | NCT04228159 | Effects of Strength Exercise on Fall Risk in Elderly With Alzheimer's Disease | 2021/7/19 | Not |
| 2 | Recruiting | Yes | NCT04061785 | Effects of physical exercise on muscle control in people with Parkinson's disease | 2021/6/1 | Not |
| 3 | Recruiting | No | TCTR20190624004 | The Effects of Weight bearing Training Combined with Elastic Band and Functional Electrical Stimulation on Gait and Balance in Stroke Patients | 2021/5/3 | Not |
| 4 | Recruiting | Yes | TCTR20190103003 | Effects of an activity-oriented physiotherapy exercise programme with and without eye movement training on dynamic balance and fall risk in people with Parkinson’s disease: a randomised controlled pilot trial | 2021/4/8 | Not |
| 5 | Recruiting | Yes | NCT02314585 | Effects of neuromobilization on upper extremity in stroke patients | 2020/9/28 | Not |
| 6 | Recruiting | Yes | ACTRN12612000224820 | Effects of prescribed Yijinjing exercise on cognitive function and fall risk in post-stroke patients: a randomized controlled trial | 2020/8/17 | Not |
| 7 | Recruiting | Yes | NCT05090774 | Effect Of strength training and fall prevention guide on balance in community dwelling elderly population | 2022/2/4 | Not |
| 8 | Recruiting | Yes | NCT04994678 | Comparison of Institutional Based Rehabilitation with Community Based Rehabilitation to prevent falls in Elderly | 2021/12/29 | Not |
| 9 | Recruiting | Yes | NCT04911179 | Feasibility and effectiveness of an online,group-based exercise program on fall, muscle strength and quality of life among older adults. | 2021/11/26 | Not |
| 10 | Recruiting | Yes | DRKS00024982 | Effectiveness of Balance exercise on Fear of Fall, Balance, and Quality of Life in Elderly. | 2021/11/24 | Not |
| 11 | Recruiting | Yes | NCT04748354 | Integrating Fall Prevention Balance Exercises Into a Program for Older Adults With Peripheral Artery Disease (PAD): A Mixed Methods Feasibility Study | 2021/10/6 | Not |
| 12 | Recruiting | Yes | NCT04759690 | Comparison of exergame and balance exercises on balance and knee proprioception of the elderly | 2021/9/14 | Not |
| 13 | Recruiting | Yes | NCT03839576 | Implementing an Evidence-based Exercise Program to Reduce Falls in Community-dwelling Older Adults (Otago) | 2021/4/9 | Not |
| 14 | Recruiting | Yes | IRCT20170120032066N2 | Does Fall Arrest Strategy Training Improve Capacity to Prevent Fall-Related Injury in Older Women? | 2021/3/8 | Not |
| 15 | Recruiting | Yes | NCT03741335 | the effect of baduanjin exercise on fall risk of older people with type II diabetese | 2021/3/5 | Not |
| 16 | Recruiting | Yes | NCT03629912 | TOE EXERCISE FOR PREVENTION FROM FALL IN ELDERLY PEOPLE | 2021/3/1 | Not |
| 17 | Recruiting | No | JPRN-UMIN000015078 | A clinical trail to study the effectiveness of balance exercise on risk of fall prevention among older people in older people in selected old age homes at Bhubaneswar, orissa.(Pilot study). | 2020/9/17 | Not |
| 18 | Recruiting | No | ACTRN12613000855729 | Effects of Core Stability Exercises on Balance and Walking in Elderly Fallers | 2020/8/19 | Not |
| 19 | Recruiting | Yes | ACTRN12609000772246 | Effect of Plantar Sensory Exercises on Balance and Fall Risk in Nursing Home Elderly | 2020/7/14 | Not |
| 20 | Recruiting | No | IRCT20180627040251N2 | The effectiveness of Thai modified fall prevention exercise on functional balance and strength, and exercise adherence in elderly with fall risks | 2021/7/28 | Not |
| 21 | Recruiting | Yes | NCT02999464 | Effects of Action Observation Training and Exercises Over 65 Years Old | 2020/12/6 | Not |

**B. ClinicalTrials.gov**

| Row | Status | Study Title | Conditions | Interventions | Locations |
| --- | --- | --- | --- | --- | --- |
| 1 | Not yet recruiting | Perturbation Training Reduces Falls in People With AD | Alzheimer Disease | Behavioral: Perturbation training |  |
| 2 | Recruiting | Multidisciplinary Home-based Tele-rehabilitation Intervention | Parkinson Disease | Procedure: Multidisciplinary tele-health intervention (physical therapy, neurologist, nurse, psychologist)  Other: Standard in-office medical care | Hospital Universitario de Burgos Burgos, Spain |
| 3 | Not yet recruiting | Effects of Multicomponent Physical Exercise Program and Mediterranean Diet in Alzheimer's Disease | Alzheimer Disease  Physical Exercise  Bone Density  Fall | Other: Multicomponent physical exercise program associated with a Mediterranean diet  Other: Usual care | University of Salamanca Salamanca, Spain |
| 4 | Recruiting | Augmented Reality Treadmill Training in Patients With Parkinson's Disease | Parkinson Disease  Fall | Device: C-Mill augmented reality treadmill training | Department of Neurology Valens, Saint Gallen, Switzerland |
| 5 | Recruiting | Highly Challenging Balance Program to Reduce Fall Rate in PD | Parkinson's Disease | Behavioral: Facility-based structured exercise  Behavioral: Home-based structured exercise  Behavioral: Health education | VA Boston Healthcare System Jamaica Plain Campus, Jamaica Plain, MA Boston, Massachusetts, United States |
| 6 | Recruiting | Protective Step Training in Parkinson's Disease | Parkinson Disease | Behavioral: Protective Step Training | Arizona State University Phoenix, Arizona, United States |

**Text S6: Included studies, n=64**

1. Arantes PMM, Dias JMD, Fonseca FF, et al. Effect of a Program Based on Balance Exercises on Gait, Functional Mobility, Fear of Falling, and Falls in Prefrail Older Women [J]. *Topics in Geriatric Rehabilitation.* 2015;31(2):113-120.

2. Ashburn A, Fazakarley L, Ballinger C, Pickering R, McLellan LD, Fitton C. A randomised controlled trial of a home based exercise programme to reduce the risk of falling among people with Parkinson's disease [J]. *J Neurol Neurosurg Psychiatry.* 2007;78(7):678-684.

3. Barnett A, Smith B, Lord SR, Williams M, Baumand A. Community-based group exercise improves balance and reduces falls in at-risk older people: a randomised controlled trial [J]. *Age Ageing.* 2003;32(4):407-414.

4. Barreca S, Sigouin, C.S., Lambert, C., and Ansley, B.,. Effects of Extra Training on the Ability of Stroke Survivors to Perform an Independent Sit-to-Stand: A Randomized Controlled Trial [J]. *Journal of Geriatric Physical Therapy.* 2004;27(2):59-64.

5. Beling J, Roller M. Multifactorial intervention with balance training as a core component among fall-prone older adults [J]. *J Geriatr Phys Ther.* 2009;32(3):125-133.

6. Beyer N, Simonsen L, Bulow J, et al. Old women with a recent fall history show improved muscle strength and function sustained for six months after finishing training [J]. *Aging Clin Exp Res* 2007;19(4):300-309.

7. Boongird C, Keesukphan P, Phiphadthakusolkul S, Rattanasiri S, Thakkinstian A. Effects of a simple home-based exercise program on fall prevention in older adults: A 12-month primary care setting, randomized controlled trial [J]. *Geriatr Gerontol Int.* 2017;17(11):2157-2163.

8. Brett L, Stapley P, Meedya S, Traynor V. Effect of physical exercise on physical performance and fall incidents of individuals living with dementia in nursing homes: a randomized controlled trial [J]. *Physiotherapy Theory and Practice.* 2019:1-14.

9. Buchner DM, Cress ME, de Lateur BJ, et al. The effect of strength and endurance training on gait, balance, fall risk, and health services use in community-living older adults [J]. *J Gerontol A Biol Sci Med Sci.* 1997;52(4):M218-224.

10. Canning CG, Sherrington C, Lord SR, et al. Exercise for falls prevention in Parkinson disease: a randomized controlled trial [J]. *Neurology.* 2015;84(3):304-312.

11. Cheng PT, Wu SH, Liaw MY, Wong AM, Tang FT. Symmetrical body-weight distribution training in stroke patients and its effect on fall prevention [J]. *Arch Phys Med Rehabil.* 2001;82(12):1650-1654.

12. Chivers Seymour K, Pickering R, Rochester L, et al. Multicentre, randomised controlled trial of PDSAFE, a physiotherapist-delivered fall prevention programme for people with Parkinson’s [J]. *Journal of Neurology, Neurosurgery & Psychiatry.* 2019;90(7):774-782.

13. Clegg A, Barber S, Young J, Iliffe S, Forster A. The Home-based Older People's Exercise (HOPE) trial: a pilot randomised controlled trial of a home-based exercise intervention for older people with frailty [J]. *Age Ageing.* 2014;43(5):687-695.

14. Clemson L, Singh MF, Bundy A, et al. LiFE Pilot Study: A randomised trial of balance and strength training embedded in daily life activity to reduce falls in older adults [J]. *Aust Occup Ther J.* 2010;57(1):42-50.

15. Clemson L, Fiatarone Singh MA, Bundy A, et al. Integration of balance and strength training into daily life activity to reduce rate of falls in older people (the LiFE study): randomised parallel trial [J]. *BMJ.* 2012;345:e4547.

16. Dadgari A, Aizan Hamid T, Hakim MN, et al. Randomized Control Trials on Otago Exercise Program (OEP) to Reduce Falls Among Elderly Community Dwellers in Shahroud, Iran [J]. *Iran Red Crescent Med J.* 2016;18(5):e26340.

17. de Souto Barreto P, Cesari M, Denormandie P, Armaingaud D, Vellas B, Rolland Y. Exercise or Social Intervention for Nursing Home Residents with Dementia: A Pilot Randomized, Controlled Trial [J]. *J Am Geriatr Soc.* 2017;65(9):E123-E129.

18. El-Khoury F, Cassou B, Latouche A, Aegerter P, Charles MA, Dargent-Molina P. Effectiveness of two year balance training programme on prevention of fall induced injuries in at risk women aged 75-85 living in community: Ossebo randomised controlled trial [J]. *BMJ.* 2015;351:h3830.

19. Elley CR, Robertson MC, Garrett S, et al. Effectiveness of a falls-and-fracture nurse coordinator to reduce falls: a randomized, controlled trial of at-risk older adults [J]. *J Am Geriatr Soc.* 2008;56(8):1383-1389.

20. Freiberger E, Haberle L, Spirduso WW, Zijlstra GA. Long-term effects of three multicomponent exercise interventions on physical performance and fall-related psychological outcomes in community-dwelling older adults: a randomized controlled trial [J]. *J Am Geriatr Soc.* 2012;60(3):437-446.

21. Gao Q, Leung A, Yang Y, et al. Effects of Tai Chi on balance and fall prevention in Parkinson's disease: a randomized controlled trial [J]. *Clin Rehabil.* 2014;28(8):748-753.

22. Goodwin VA, Richards SH, Henley W, Ewings P, Taylor AH, Campbell JL. An exercise intervention to prevent falls in people with Parkinson's disease: a pragmatic randomised controlled trial [J]. *J Neurol Neurosurg Psychiatry.* 2011;82(11):1232-1238.

23. Haines TP, Russell T, Brauer SG, et al. Effectiveness of a video-based exercise programme to reduce falls and improve health-related quality of life among older adults discharged from hospital: a pilot randomized controlled trial [J]. *Clin Rehabil.* 2009;23(11):973-985.

24. Hauer K, Rost B, Rutschle K, et al. Exercise training for rehabilitation and secondary prevention of falls in geriatric patients with a history of injurious falls [J]. *J Am Geriatr Soc.* 2001;49(1):10-20.

25. Hirase T, Inokuchi S, Matsusaka N, Okita M. Effects of a balance training program using a foam rubber pad in community-based older adults: a randomized controlled trial [J]. *J Geriatr Phys Ther.* 2015;38(2):62-70.

26. Iliffe S, Kendrick D, Morris R, et al. Multicentre cluster randomised trial comparing a community group exercise programme and home-based exercise with usual care for people aged 65 years and over in primary care [J]. *Health Technol Assess.* 2014;18(49):vii-xxvii, 1-105.

27. Kim H, Yoshida H, Suzuki T. Falls and fractures in participants and excluded non-participants of a fall prevention exercise program for elderly women with a history of falls: 1-year follow-up study [J]. *Geriatr Gerontol Int.* 2014;14(2):285-292.

28. Kovacs E, Sztruhar Jonasne I, Karoczi CK, Korpos A, Gondos T. Effects of a multimodal exercise program on balance, functional mobility and fall risk in older adults with cognitive impairment: a randomized controlled single-blind study [J]. *Eur J Phys Rehabil Med.* 2013;49(5):639-648.

29. Kruse RL, Lemaster JW, Madsen RW. Fall and balance outcomes after an intervention to promote leg strength, balance, and walking in people with diabetic peripheral neuropathy: "feet first" randomized controlled trial [J]. *Phys Ther.* 2010;90(11):1568-1579.

30. Latham NK, Anderson CS, Lee A, et al. A randomized, controlled trial of quadriceps resistance exercise and vitamin D in frail older people: the Frailty Interventions Trial in Elderly Subjects (FITNESS) [J]. *J Am Geriatr Soc.* 2003;51(3):291-299.

31. Li F, Harmer P, Fitzgerald K, et al. Tai chi and postural stability in patients with Parkinson's disease [J]. *N Engl J Med.* 2012;366(6):511-519.

32. Lin MR, Wolf SL, Hwang HF, Gong SY, Chen CY. A randomized, controlled trial of fall prevention programs and quality of life in older fallers [J]. *J Am Geriatr Soc.* 2007;55(4):499-506.

33. Liu-Ambrose T, Donaldson MG, Ahamed Y, et al. Otago home-based strength and balance retraining improves executive functioning in older fallers: a randomized controlled trial [J]. *J Am Geriatr Soc.* 2008;56(10):1821-1830.

34. Liu-Ambrose T, Davis JC, Best JR, et al. Effect of a Home-Based Exercise Program on Subsequent Falls Among Community-Dwelling High-Risk Older Adults After a Fall: A Randomized Clinical Trial [J]. *JAMA.* 2019;321(21):2092-2100.

35. Logghe IH, Zeeuwe PE, Verhagen AP, et al. Lack of effect of Tai Chi Chuan in preventing falls in elderly people living at home: a randomized clinical trial [J]. *J Am Geriatr Soc.* 2009;57(1):70-75.

36. Luukinen H, Lehtola S, Jokelainen J, Vaananen-Sainio R, Lotvonen S, Koistinen P. Pragmatic exercise-oriented prevention of falls among the elderly: a population-based, randomized, controlled trial [J]. *Prev Med.* 2007;44(3):265-271.

37. Mahoney JE, Shea TA, Przybelski R, et al. Kenosha County falls prevention study: a randomized, controlled trial of an intermediate-intensity, community-based multifactorial falls intervention [J]. *J Am Geriatr Soc.* 2007;55(4):489-498.

38. Marigold DS, Eng JJ, Dawson AS, Inglis JT, Harris JE, Gylfadottir S. Exercise leads to faster postural reflexes, improved balance and mobility, and fewer falls in older persons with chronic stroke [J]. *J Am Geriatr Soc.* 2005;53(3):416-423.

39. Morgan RO, Virnig BA, Duque M, Abdel-Moty E, Devito CA. Low-intensity exercise and reduction of the risk for falls among at-risk elders [J]. *J Gerontol A Biol Sci Med Sci.* 2004;59(10):1062-1067.

40. Morris ME, Menz HB, McGinley JL, et al. A Randomized Controlled Trial to Reduce Falls in People With Parkinson's Disease [J]. *Neurorehabil Neural Repair.* 2015;29(8):777-785.

41. Nitz JC, Choy NL. The efficacy of a specific balance-strategy training programme for preventing falls among older people: a pilot randomised controlled trial [J]. *Age Ageing.* 2004;33(1):52-58.

42. Nyman SR, Ingram W, Sanders J, et al. Randomised Controlled Trial Of The Effect Of Tai Chi On Postural Balance Of People With Dementia</p> [J]. *Clinical Interventions in Aging.* 2019;Volume 14:2017-2029.

43. Ohman H, Savikko N, Strandberg T, et al. Effects of Exercise on Functional Performance and Fall Rate in Subjects with Mild or Advanced Alzheimer's Disease: Secondary Analyses of a Randomized Controlled Study [J]. *Dement Geriatr Cogn Disord.* 2016;41(3-4):233-241.

44. Pang MYC, Yang L, Ouyang H, Lam FMH, Huang M, Jehu DA. Dual-Task Exercise Reduces Cognitive-Motor Interference in Walking and Falls After Stroke [J]. *Stroke.* 2018;49(12):2990-2998.

45. Patil R, Uusi-Rasi K, Tokola K, Karinkanta S, Kannus P, Sievanen H. Effects of a Multimodal Exercise Program on Physical Function, Falls, and Injuries in Older Women: A 2-Year Community-Based, Randomized Controlled Trial [J]. *J Am Geriatr Soc.* 2015;63(7):1306-1313.

46. Pitkälä K, Savikko N, Poysti M, Strandberg T, Laakkonen M-L. Efficacy of physical exercise intervention on mobility and physical functioning in older people with dementia: A systematic review [J]. *Experimental Gerontology.* 2013;48(1):85-93.

47. Puente-González AS, Sánchez-Sánchez MC, Fernández-Rodríguez EJ, Hernández-Xumet JE, Barbero-Iglesias FJ, Méndez-Sánchez R. Effects of 6-Month Multimodal Physical Exercise Program on Bone Mineral Density, Fall Risk, Balance, and Gait in Patients with Alzheimer’s Disease: A Controlled Clinical Trial [J]. *Brain Sciences.* 2021;11(1).

48. Rolland Y, Pillard F, Klapouszczak A, et al. Exercise program for nursing home residents with Alzheimer's disease: a 1-year randomized, controlled trial [J]. *J Am Geriatr Soc.* 2007;55(2):158-165.

49. Rosendahl E, Gustafson Y, Nordin E, Lundin-Olsson L, Nyberg L. A randomized controlled trial of fall prevention by a high-intensity functional exercise program for older people living in residential care facilities [J]. *Aging Clin Exp Res.* 2008;20(1):67-75.

50. Sakamoto K, Nakamura T, Hagino H, et al. Effects of unipedal standing balance exercise on the prevention of falls and hip fracture among clinically defined high-risk elderly individuals: a randomized controlled trial [J]. *J Orthop Sci.* 2006;11(5):467-472.

51. Sakamoto K, Endo N, Harada A, et al. Why not use your own body weight to prevent falls? A randomized, controlled trial of balance therapy to prevent falls and fractures for elderly people who can stand on one leg for </=15 s [J]. *J Orthop Sci.* 2013;18(1):110-120.

52. Salminen MJ, Vahlberg TJ, Salonoja MT, Aarnio PT, Kivela SL. Effect of a risk-based multifactorial fall prevention program on the incidence of falls [J]. *J Am Geriatr Soc.* 2009;57(4):612-619.

53. Sherrington C, Lord SR, Vogler CM, et al. A post-hospital home exercise program improved mobility but increased falls in older people: a randomised controlled trial [J]. *PLoS One.* 2014;9(9):e104412.

54. Siegrist M, Freiberger E, Geilhof B, et al. Fall Prevention in a Primary Care Setting [J]. *Deutsches Aerzteblatt Online.* 2016.

55. Skelton D, Dinan S, Campbell M, Rutherford O. Tailored group exercise (Falls Management Exercise -- FaME) reduces falls in community-dwelling older frequent fallers (an RCT) [J]. *Age Ageing.* 2005;34(6):636-639.

56. Smulders E, Weerdesteyn V, Groen BE, et al. Efficacy of a short multidisciplinary falls prevention program for elderly persons with osteoporosis and a fall history: a randomized controlled trial [J]. *Arch Phys Med Rehabil.* 2010;91(11):1705-1711.

57. Sparrow D, DeAngelis TR, Hendron K, Thomas CA, Saint-Hilaire M, Ellis T. Highly Challenging Balance Program Reduces Fall Rate in Parkinson Disease [J]. *Journal of Neurologic Physical Therapy.* 2016;40(1):24-30.

58. Steadman J, Donaldson N, Kalra L. A randomized controlled trial of an enhanced balance training program to improve mobility and reduce falls in elderly patients [J]. *J Am Geriatr Soc.* 2003;51(6):847-852.

59. Taylor-Piliae RE, Hoke TM, Hepworth JT, Latt LD, Najafi B, Coull BM. Effect of Tai Chi on physical function, fall rates and quality of life among older stroke survivors [J]. *Arch Phys Med Rehabil.* 2014;95(5):816-824.

60. Toots A, Wiklund R, Littbrand H, et al. The Effects of Exercise on Falls in Older People With Dementia Living in Nursing Homes: A Randomized Controlled Trial [J]. *J Am Med Dir Assoc.* 2018.

61. Uusi-Rasi K, Patil R, Karinkanta S, et al. Exercise and vitamin D in fall prevention among older women: a randomized clinical trial [J]. *JAMA Intern Med.* 2015;175(5):703-711.

62. Wesson J, Clemson L, Brodaty H, et al. A feasibility study and pilot randomised trial of a tailored prevention program to reduce falls in older people with mild dementia [J]. *BMC Geriatr.* 2013;13:89.

63. Wolf SL, Barnhart HX, Kutner NG, McNeely E, Coogler C, Xu T. Reducing frailty and falls in older persons: an investigation of Tai Chi and computerized balance training. Atlanta FICSIT Group. Frailty and Injuries: Cooperative Studies of Intervention Techniques [J]. *J Am Geriatr Soc.* 1996;44(5):489-497.

64. Yang XJ, Hill K, Moore K, et al. Effectiveness of a targeted exercise intervention in reversing older people's mild balance dysfunction: a randomized controlled trial [J]. *Phys Ther.* 2012;92(1):24-37.
